# Frequency‐dependent two‐sex models: a new approach to sex ratio evolution with multiple maternal conditions

**DOI:** 10.1002/ece3.2202

**Published:** 2016-09-07

**Authors:** Esther Shyu, Hal Caswell

**Affiliations:** ^1^ Biology Department MS‐34 Woods Hole Oceanographic Institution Woods Hole Massachusetts 02543; ^2^ Institute for Biodiversity and Ecosystem Dynamics University of Amsterdam Amsterdam The Netherlands

**Keywords:** Adaptive dynamics, facultative sex ratios, maternal condition, matrix population models, sex ratio evolution, Trivers‐Willard hypothesis

## Abstract

Mothers that experience different individual or environmental conditions may produce different proportions of male to female offspring. The Trivers‐Willard hypothesis, for instance, suggests that mothers with different qualities (size, health, etc.) will use different sex ratios if maternal quality differentially affects sex‐specific reproductive success. Condition‐dependent, or facultative, sex ratio strategies like these allow multiple sex ratios to coexist within a population. They also create complex population structure due to the presence of multiple maternal conditions. As a result, modeling facultative sex ratio evolution requires not only sex ratio strategies with multiple components, but also two‐sex population models with explicit stage structure. To this end, we combine nonlinear, frequency‐dependent matrix models and multidimensional adaptive dynamics to create a new framework for studying sex ratio evolution. We illustrate the applications of this framework with two case studies where the sex ratios depend one of two possible maternal conditions (age or quality). In these cases, we identify evolutionarily singular sex ratio strategies, find instances where one maternal condition produces exclusively male or female offspring, and show that sex ratio biases depend on the relative reproductive value ratios for each sex.

## Introduction

The primary sex ratio can be defined as the proportion of male births in an individual's offspring production strategy (Trivers 1985). When the primary sex ratio is homogenous across the population, it evolves to equalize parental investment in both sexes (Fisher 1930). If males and females are equally costly, the sex ratio evolves to equality (Hamilton 1967). If males and females are differentially costly, the sex ratio skews in response to sex‐specific offspring costs, such as differential offspring resource requirements, offspring mortality, or offspring‐induced parental mortality (Shyu and Caswell 2016).

However, many species have facultative (condition‐dependent) sex ratio strategies, where a parent adjusts the primary sex ratio of its offspring depending on some environmental or individual condition (Leimar 1996; West 2009). These facultative sex ratio strategies allow both multiple mating stages and multiple sex ratios to coexist within a population.

In order to better incorporate these factors into evolutionary projections, we have developed a two‐sex modeling framework that has multiple maternal states with different sex ratios. Our general model is introduced in the section “A two‐sex matrix model with multiple maternal conditions” and further expanded upon in the “Model” sections of two case studies.

This framework combines three components that have never (to our knowledge) been simultaneously applied to the problem of facultative sex ratio evolution. We include arbitrary stage structures within male and female life cycles. We make the demographic model nonlinear, to include the dependence of reproductive success on the stage‐sex composition of the population; this dependence provides a route through which sex ratio strategies will feed back on the fitness of the individuals adopting them, which is largely ignored in the current literature (e.g., Pen et al. 1999; Fawcett et al. 2011; Schindler et al. 2015). Finally, rather than relying on traditional criteria for sex ratio evolution that were derived for simpler cases (e.g., Schindler et al. 2015), we make use of the explicit evolutionary calculations obtained from adaptive dynamics. Thus, our modeling framework relaxes three of the primary simplifying assumptions that are common in the literature on sex ratio evolution.

To illustrate our framework, we will focus on a situation with two conditions of mothers, so that the facultative sex ratio strategy is described by the bivariate trait vector **s**:

(1) $$s=\left(\begin{array}{c} s_{1}\\ s_{2} \end{array}\right)$$

where $s_{1}$ is the sex ratio used by mothers in one condition (e.g., low quality) and $s_{2}$ is the sex ratio used by mothers in the other condition (e.g., high quality). Using multidimensional adaptive dynamics methods, as described in the section “Multidimensional adaptive dynamics”, we determine how **s** evolves over time and find its evolutionarily singular strategies $s^{}*$, which are potential long‐term evolutionary outcomes.

We then consider two specific cases where the sex ratio depends on maternal condition. In the first case (“Case 1: Maternal age”), young and old mothers can evolve different sex ratios. In the second case (“Case 2: Maternal quality”), high‐ and low‐quality mothers can evolve different sex ratios, as in the Trivers‐Willard hypothesis.

## Background

Many species adjust the sex ratios of their offspring in response to spatial or temporal environmental variation. Parasitic wasps, which lay their eggs on a variety of hosts, vary their sex ratios based on host size (Charnov et al. 1981); because female larvae benefit more from larger food sources, wasp sex ratios are female‐biased on large hosts and male‐biased on small ones. Other species use different sex ratios in different seasons, in response to the timing of sex‐specific life cycle events (Werren and Charnov 1978). Kestrels, for instance, produce offspring at different sex ratios at different points in the breeding season, to account for male and female maturation differences (Pen et al. 1999).

Sex ratios may also vary with some parental (usually maternal) condition, such as age. In many mammals, where males have higher infant mortality rates (Trivers 1985), sex ratios become increasingly female‐biased with maternal age. This may be because older mothers are more prone to death or sterility and cannot replace lost sons as easily (Charlesworth 1977). Older fathers can also promote female‐biased sex ratios; *Drosophila melanogaster* females with older mates tend to produce more female offspring, possibly because deleterious mutations in older fathers are more detrimental to sons (Long and Pischedda 2005). When older parents are more beneficial, sex ratio biases reverse. In Iberian red deer, for example, older females are larger, obtain more food, and expend more energy on reproduction. Sex ratios thus become increasingly male‐biased with maternal age, because older mothers can afford the higher costs of sons (Lendete‐Castillejos et al. 2004).

Other parental conditions, including health, size, or ranking, affect the conditions of the parent's offspring (Hewison and Gaillard 1999). These types of conditions, or qualities, are the focus of the Trivers‐Willard hypothesis (Trivers and Willard 1973). The Trivers‐Willard hypothesis predicts that a parent's quality will affect the sex ratios of their offspring if offspring quality is correlated with parental quality. Namely, low‐quality parents should preferentially produce offspring of the sex with higher reproductive success (e.g., number of offspring) at low quality, and vice versa for high‐quality parents.

**Figure 1:**
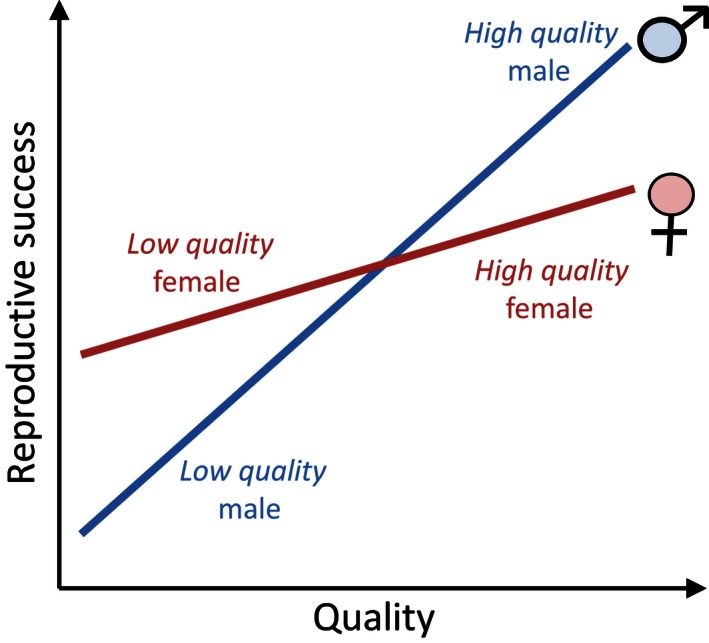
Reproductive success as a function of quality in the Trivers‐Willard hypothesis. In this example, low‐quality females are more successful than low‐quality males, but high‐quality males and more successful than high‐quality females.

Suppose that reproductive success of male offspring varies more steeply with quality than that of female offspring. Then, low‐quality female offspring will have higher success than low‐quality male offspring, but this ranking will be reversed for high‐quality offspring (Fig. 1). Thus, low‐quality parents, doomed to produce low‐quality offspring, should favor females. High‐quality parents, anticipating the production of high‐quality offspring, should favor males. The influence of quality on reproductive success is usually described in terms of greater (males, in our example) or lesser (females) “variance in reproductive success,” and the Trivers‐Willard hypothesis is usually phrased as a prediction that high‐quality mothers will invest more in the sex with greater variance in reproductive output.

The example with males and females we used here is often applied to polygynous species, where high‐quality males monopolize most breeding opportunities. As a result, a high‐quality son outreproduces a high‐quality daughter, but a low‐quality daughter outreproduces a low‐quality son. Because males are the sex with the greater variance in reproductive success, high‐quality females adjust their sex ratios to invest in sons, while low‐quality females invest in daughters.

Empirical support for the Trivers‐Willard hypothesis has been found in many animals, most notably ungulates with strong sexual dimorphism and polygynous mating (e.g., Clutton‐Brock et al. 1984, 1986; Hewison and Gaillard 1999). However, results are sometimes mixed, possibly due to differences in data collection methods, quality measures, and difficulties in calculating lifetime reproductive success (e.g., Hewison and Gaillard 1999; Sheldon and West 2004; Schindler et al. 2015). The effects of maternal quality on sex ratio have also been studied in birds (e.g., Kilner 1998; Clout et al. 2002), humans (e.g., Gaulin and Robbins 1991; Cameron and Dalerum 2009), and many other taxa (West 2009, Ch. 6)

## Model and Methods

Here, we present a framework for modeling facultative sex ratio evolution, one that includes multiple maternal conditions such as ages and the qualities described in the Trivers‐Willard hypothesis. The basis of our framework is a two‐sex population model that uses a series of matrices to describe various stages and life cycle processes (as in Shyu and Caswell 2016; Shyu and Caswell Submitted manuscript). Our model distinguishes between two individual conditions (e.g., age or quality) for both males and females. We accordingly incorporate four types of unions (male–female mated pairings) and different preferences for mating with partners in different conditions.

Each maternal condition produces offspring with a different sex ratio. We analyze the transient dynamics of bivariate sex ratio evolution using the canonical equation of adaptive dynamics and the equilibrium dynamics by characterizing evolutionarily singular strategies (SSs) of the sex ratios.

### A two‐sex matrix model with multiple maternal conditions

Consider, as an example, a two‐sex population consisting of Condition 1 and Condition 2 individuals (e.g., young and old individuals, low‐ and high‐quality individuals). Males and females mate to form unions (here, monogamous couples) that produce new offspring.

Unions where the male partner is in Condition *i* and the female partner is in Condition *j* will be written as $u_{ij}$. As we shall see in the “Model” sections for the two case studies, the $u_{ij}$ may differ in available resources, fertilities, and other properties. We will specifically assume that any union with a Condition *j* female has sex ratio $s_{j}$. This means that the primary sex ratio is a facultative trait that depends solely on maternal condition.

**Figure 2:**
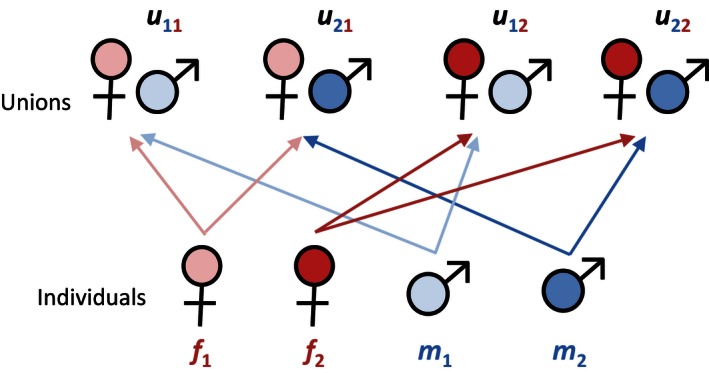
Stages of unmated individuals and mated unions in a two‐sex population. Both males and females have two possible conditions.

The population consists of conditions 1 and 2 males ($m_{1},m_{2}$), conditions 1 and 2 females ($f_{1},f_{2}$), and four types of unions (Fig. 2). The densities of each stage are given by the population vector:

(2) $$n\left(t\right)=\left(\begin{array}{c} m_{1}\\ m_{2}\\ f_{1}\\ f_{2}\\ u_{11}\\ u_{21}\\ u_{12}\\ u_{22} \end{array}\right)=\left(\begin{array}{c} \text{Condition 1 males}\\ \text{Condition 2 males}\\ \text{Condition 1 females}\\ \text{Condition 2 females}\\ \text{Condition 1 male + Condition 1 female}\\ \text{Condition 2 male + Condition 1 female}\\ \text{Condition 1 male + Condition 2 female}\\ \text{Condition 2 male + Condition 2 female} \end{array}\right)$$

Additional male, female, or union types can be added as new entries in the population vector.

We divide mating, birth, and life cycle transition processes into three rate matrices: **U**,** B**, and **T**, respectively. As shown in Shyu and Caswell (Submitted manuscript), the average of these matrices is the continuous‐time projection matrix:

(3) $$A\left(n\right)=\frac{1}{3}\left[T+B+U\right]$$

where

(4) $$\frac{dn}{dt}=A\left(n\right)n\left(t\right)$$

Specific examples of both the population vector and rate matrices are given in the “Model” sections for the two case studies. As we shall see, each of the three rate matrices in (3) may depend on the population vector (2) or the sex ratio vector (1)

The essential property of two‐sex models is that, in sexually reproducing species, reproduction depends on the *relative* abundance of males and females, or of males and females of particular life cycle stages. In the extreme case, as the relative abundance of either stage declines to zero, reproductive success also declines to zero. In less extreme imbalances of the sex structure, reproduction will still be affected by the availability of mates, in a way that depends on the life history and mating system of the species.

Thus, the matrices in (3) are functions of the stage frequency vector:

(5) $$p=\frac{n}{\left\|n\right\|}$$

where ‖**n**‖ is the 1‐norm of **n**. As a result, (4) is a frequency‐dependent model that converges to an equilibrium stage distribution $\hat{p}$ and a growth rate *λ* that is the dominant eigenvalue of $A(\hat{p})$. This is a general property of frequency‐dependent models (Ianelli et al. 2005). To calculate *λ* and $\hat{p}$, it is sufficient to consider the dynamics of **p** (Shyu and Caswell Submitted manuscript):

(6) $$\frac{dp}{dt}=\left(I_{s}-p1^{⊺}\right)A\left(p\right)p$$

To find $\hat{p}$, we integrate (6) with the MATLAB ODE45 differential equation solver until **p** converges to $\hat{p}$ (e.g., until vector entries do not change significantly over consecutive integration intervals). The population's long‐term growth rate *λ* is then the dominant eigenvalue of $A(\hat{p})$, which has corresponding right and left eigenvectors **w** and **v**. Note that the right eigenvector is also the stable stage distribution; that is, $w=\hat{p}$.

### Mating preferences

The mating process, where adult males and females pair into reproducing unions, is described by the union formation matrix **U**. Mating functions in **U** give the rates of union formation as functions of the relative frequencies of males and females available to mate and are thus functions of the stage frequency vector $\hat{p}$ (5).

Mating preferences in the mating functions describe the probabilities of favoring partners of certain conditions. The female preference distribution $g_{j}\left(i\right)$ gives the proportion of Condition *j* females that mate with Condition *i* males. Similarly, the male preference distribution $h_{i}\left(j\right)$ gives the proportion of Condition *i* males that mate with Condition *j* females. Summing these distributions over all male and female conditions, respectively, yields a total probability of 1:

(7) $$\sum_{i}g_{j}\left(i\right)=1\;\forall \;j$$

(8) $$\sum_{j}h_{i}\left(j\right)=1\;\forall \;i$$

Examples of mating preference distributions include:

1. *Fully assortative mating*, where individuals only mate with partners in the same condition:
  (9) $$\begin{array}{cc} g_{j}\left(i\right) & =1\;\;\text{if}\;i=j,0\;\text{else}\\ h_{i}\left(j\right) & =1\;\;\text{if}\;i=j,0\;\text{else}\; \end{array}$$
2. *Random mating*, where individuals pick partners based on their relative abundances in the population:
  (10) $$\begin{array}{cc} g_{j}\left(i\right) & =\frac{m_{i}}{\sum_{i}m_{i}}\\ h_{i}\left(j\right) & =\frac{f_{j}}{\sum_{j}f_{j}} \end{array}$$
3. *Biased mating*, where individuals prefer partners of certain conditions. An attractiveness or competitiveness factor $c_{i}$ weighs the abundance of each partner condition, for example:
  (11) $$\begin{array}{cc} g_{j}\left(i\right) & =\frac{c_{i}m_{i}}{\sum_{i}c_{i}m_{i}}\\ h_{i}\left(j\right) & =\frac{c_{j}f_{j}}{\sum_{j}c_{j}f_{j}} \end{array}$$
  Partners with larger $c_{i}$ are more preferable mates. If all $c_{i}$ are equal, (11) reduces to the random mating case (10). If $c_{i}=0$, individuals of stage *i* do not mate.

The total mating function $M_{ij}\left(n\right)$ gives the total unions $u_{ij}$ (Condition *i* males mated with Condition *j* females) formed per time. The most general and flexible mating functions are based on generalized weighted means (Hölder means). These have the general form:

(12) $$M_{ij}\left(n\right)={\left(b{\left[f_{j}g_{j}\left(i\right)\right]}^{a}+\left(1-b\right){\left[m_{i}h_{i}\left(j\right)\right]}^{a}\right)}^{\frac{1}{a}}$$

where 0 ≤ *b* ≤ 1 and *a* < 0 (Hadeler 1989; Caswell 2001; Martcheva and Milner 2001). Note that $M_{ij}\left(n\right)$ is calculated only over individuals that are available to mate (i.e., adult single male stages $m_{i}$ and adult single female stages $f_{j}$). As a result, the mating function does not depend on the males and females in nonmating stages, such as immature juveniles or adults already in unions.

The harmonic mean mating function in particular is one of the most widely used mating functions, because it satisfies the biological criteria for two‐sex models and is typical of a wide range of Holder means (Caswell and Weeks 1986, Ianelli et al. 2005). Here, as in Shyu and Caswell (Submitted manuscript), we use a harmonic mean mating function where *a* = −1, *b* = 1/2, so that:

(13) $$M_{ij}\left(n\right)=\frac{2m_{i}h_{i}\left(j\right)f_{j}g_{j}\left(i\right)}{m_{i}h_{i}\left(j\right)+f_{j}g_{j}\left(i\right)}$$

The corresponding male and female per capita mating functions are:

(14) $$\begin{array}{cc} U_{m,ij}\left(n\right) & =\frac{M_{ij}\left(n\right)}{m_{i}}\\ U_{f,ij}\left(n\right) & =\frac{M_{ij}\left(n\right)}{f_{j}} \end{array}$$

As we shall see, the union matrix **U** from (4) contains these per capita mating functions.

### Multidimensional adaptive dynamics

Adaptive dynamics is a phenotype‐based framework for modeling evolution. We have previously used univariate (one‐dimensional) adaptive dynamics to determine evolutionarily singular strategies for a single, scalar sex ratio (Shyu and Caswell 2016). Here, we use multidimensional adaptive dynamics to analyze the evolution of the bivariate sex ratio **s** in (1).

Similar to the approach in Shyu and Caswell (2016), we consider a stable, monomorphic resident population with sex ratio phenotype **s**, projection matrix **A** as in (3), and a long‐term exponential growth rate *λ* that is the dominant eigenvalue of $A(\hat{p})$. This resident population is invaded by new, rare mutants, which differ from residents only in terms of their sex ratio phenotype. Such mutations are small, rare, and infrequent. As a result, mutants do not affect resident dynamics and will either die out or reach fixation before the next mutation arises (Gertiz et al. 1998; Metz 2006).

To successfully invade, the mutant strategy must be able to outperform the resident, under the conditions created by the resident. A given mutant has phenotype $s^{\prime }$, projection matrix $A^{\prime }$, and corresponding growth rate $\lambda^{\prime }$; both $A^{\prime }$ and $\lambda^{\prime }$ depend on the environmental conditions (e.g., mating rates) set by the resident. The mutant projection matrix $A^{\prime }$ is structurally identical to the resident matrix **A**; however, $A^{\prime }$ uses the mutant sex ratio $s^{\prime }$ and is evaluated at the resident equilibrium stage distribution $\hat{p}$. An example of how to construct $A^{\prime }$ is shown in Shyu and Caswell 2016, section 3.2.1)

The invasion fitness $\Lambda_{s}\left(s^{\prime }\right)$ is the relative growth rate of a mutant with sex ratio strategy $s^{\prime }$, in an environment where the resident uses the strategy **s**:

(15) $$\Lambda_{s}\left(s^{\prime }\right)=\lambda^{\prime }\left(\hat{p}\right)-\lambda$$

Only mutants with a positive invasion fitness have a positive probability of displacing the resident.

The selection gradient is the first derivative of the invasion fitness (15) with respect to the mutant phenotype $s^{\prime }$, and indicates the direction of selection at a resident phenotype **s**. Note that the resident growth rate *λ* does not depend on $s^{\prime }$. Thus, the selection gradient is simply the sensitivity of mutant growth rate $\lambda^{\prime }$ (Caswell 2010):

(16) $$\begin{array}{cc} D(s) & =\frac{\partial \lambda^{\prime }}{\partial {s^{\prime }}^{⊺}}{|}_{s^{\prime }=s}\\ & =\left(\left(w^{\prime }{}^{⊺}⊗v^{\prime }{}^{⊺}\right)\frac{\text{dvec}\;A^{\prime }}{d{s^{\prime }}^{⊺}}\right){|}_{s^{\prime }=s} \end{array}$$

where $w^{\prime }$ and $v^{\prime }$ are the dominant right and left eigenvectors of the mutant matrix $A^{\prime }\left(\hat{p}\right)$, scaled so that ${v^{\prime }}^{⊺}w^{\prime }=1$.

Although the invasion fitness (15) is a scalar, the selection gradient (16) is a row vector with two components – the partial derivatives of $\lambda^{\prime }$ to each entry of **s** (1):

(17) $$D\left(s\right)=\frac{\partial \lambda^{\prime }}{\partial {s^{\prime }}^{⊺}}{|}_{s^{\prime }=s}=\left(\frac{\partial \lambda^{\prime }}{\partial s_{1}^{\prime }}{|}_{s^{\prime }=s}\;\frac{\partial \lambda^{\prime }}{\partial s_{2}^{\prime }}{|}_{s^{\prime }=s}\right)$$

As shown in the next two sections, the selection gradient (17) lends insight into both the transient and equilibrium evolutionary dynamics of **s**.

#### Evolutionary dynamics

The transient dynamics of **s** depend on the evolutionary trajectories generated by repeated mutant invasions. When mutations are small (do not differ drastically from the resident phenotype), these trajectories can be approximated by the canonical equation of adaptive dynamics.

As shown by Dieckmann and Law (1996), Durinx et al. (2005) and Durinx et al. (2008), the canonical equation is a differential equation that describes d**s**/d*t*, the change in the resident trait over time, using a first‐order Taylor approximation. In both unstructured and structured populations, it can be written as the product of the selection gradient **D**(**s**) and a mutational variance–covariance matrix **V**(**s**) that encompasses mutation probabilities, frequencies, and effects (Doebeli 2011):

(18) $$\frac{ds}{dt}=V\left(s\right)D^{⊺}\left(s\right)$$

The multivariate breeder's equation from quantitative genetics (Lande 1979) has a form similar to (18), but is based on standing genetic variation rather than the active mutational process (Doebeli 2011).

Although population size affects the mutation rate (Dieckmann and Law 1996), we will focus on the shape and direction of the evolutionary trajectories, rather than their speed, so that the population's (exponentially growing) size is irrelevant. We will also assume that effects of mutations on different components of **s** are uncorrelated (i.e., no pleiotropy), so that **V**(**s**) is a diagonal matrix.

The evolution of **s** is biologically constrained, in that neither $s_{1}$ nor $s_{2}$ can be <0 or >1 (or both 0 and 1 simultaneously) in a realistic, viable population. These constraints can be written as follows:

(19) $$\begin{array}{cc} & 0\leq s_{1}\leq 1\\ & 0\leq s_{2}\leq 1\\ & \left(s_{1},s_{2}\right)\neq \left(0,0\right)\\ & \left(s_{1},s_{2}\right)\neq \left(1,1\right) \end{array}$$

Because we are interested in the direction and outcome of evolution, but not its speed, we can solve the boundary problems by adjusting the variance–covariance matrix to prevent evolution in unfeasible directions (Dieckmann et al. 2006). To this end, we use a mutational matrix of the form:

(20) $$V\left(s\right)=\left(\begin{array}{cc} s_{1}\left(1-s_{1}\right) & 0\\ 0 & s_{2}\left(1-s_{2}\right) \end{array}\right)$$

This choice of **V** causes the mutational variances to decrease as $s_{1}$ and $s_{2}$ near 0 or 1. If either $s_{1}$ or $s_{2}$ goes to 0 or 1, their corresponding component of the canonical equation (18) will vanish, preventing that sex ratio from evolving out of the biologically constrained region (19).

With mutational matrix (20) and selection gradient (17), the canonical equation (18) becomes:

(21) $$\begin{array}{cc} \frac{ds}{dt}= & \;V\left(s\right)D^{⊺}\left(s\right)\\ = & \;\left(\begin{array}{cc} s_{1}\left(1-s_{1}\right) & 0\\ 0 & s_{2}\left(1-s_{2}\right) \end{array}\right)\left(\begin{array}{c} \frac{\partial \lambda^{\prime }}{\partial s_{1}}{|}_{s^{\prime }=s}\\ \frac{\partial \lambda^{\prime }}{\partial s_{2}}{|}_{s^{\prime }=s} \end{array}\right) \end{array}$$

We will use (21) to track the evolutionary trajectories of **s** through 2D trait space.

#### Equilibrium evolutionary dynamics

Potential evolutionary endpoints occur at stationary points of the canonical equation (21). The corresponding resident strategies $s^{}*$ are called singular strategies (SSs), where:

(22) $$\frac{ds}{dt}{|}_{s^{\prime }=s=s^{}*}=V\left(s\right)D^{⊺}\left(s\right){|}_{s^{\prime }=s=s^{}*}=0$$

**Figure 3:**
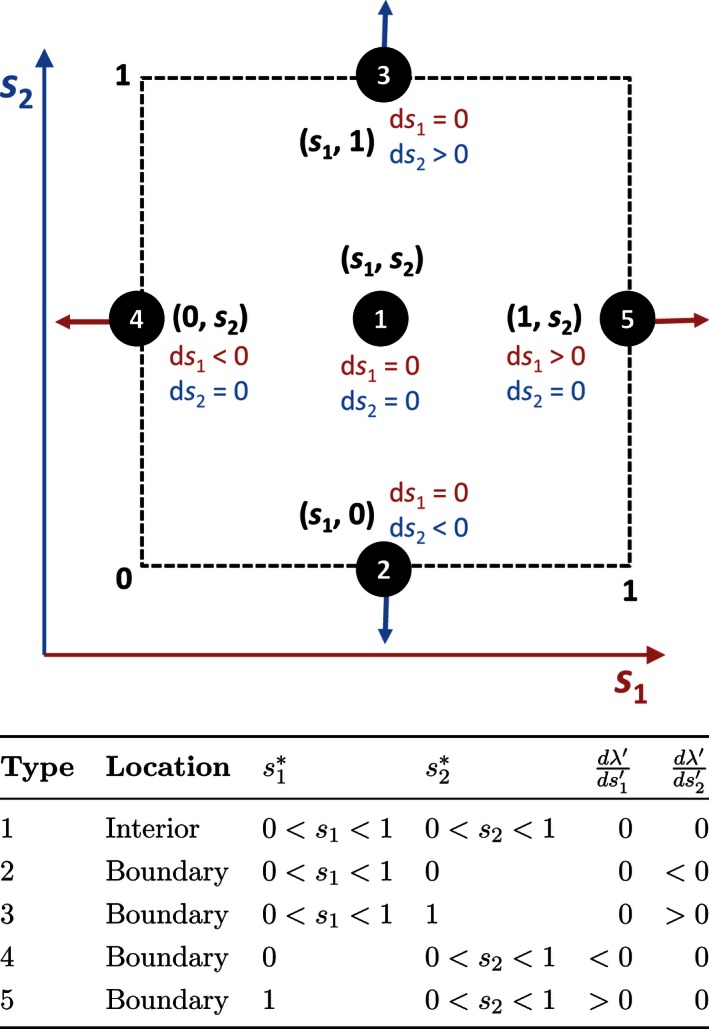
The five types of singular strategies $s^{}*=\left(s_{1}^{}*,s_{2}^{}*\right)$ and their corresponding selection gradients (17). Although it is also possible for $s^{}*=\left(0,1\right)$ or (1,0), these are marginal cases that we have not observed in our model.

As summarized in Figure 3, there are five possible types of singular strategies. The most obvious type of singular strategy (Type 1, interior SS) occurs when both entries of the selection gradient **D**(**s**) (17) are simultaneously 0, indicating no directional selection on either component of **s** (Doebeli 2011). If there are no points in the biologically constrained region (19) where both entries of **D**(**s**) are 0, there is no interior SS.

The remaining types of singular strategies (Types 2–5, boundary SSs) lie on each of the four boundaries of the constrained region, where $s_{1}$ or $s_{2}$ are either 0 or 1 (Leimar 1996; Schwanz et al. 2006). In these cases, both components of **D**(**s**) do not simultaneously equal 0. Instead, the selection gradient for the nonboundary sex ratio is 0, and the selection gradient for the boundary sex ratio points away from the boundary (Fig. 3). Note that (22) can still be satisfied depending on the value of **V**(**s**).

In most cases, as shown in Schwanz et al. (2006), there is a single SS $s^{}*$, which falls into one of these five cases (but see the “Results” section for “Case 1b: Parental resource cost” for an example where this is not true). To find $s^{}*$, we use the following steps:

1. Determine if there is any point in the constrained region (19) where both components of the selection gradient (17) are simultaneously 0. This can be done using MATLAB's fsolve or fmincon functions. If a solution is found, this is an interior (Type 1) SS. Else, we must check for a boundary SSs of Types 2–5.
2. To check for a Type 2 SS, set $s_{2}\;=\;0$ and use MATLAB's fsolve function to find the corresponding value of $s_{1}$ where $\frac{d\lambda }{ds_{1}^{\prime }}\;=\;0$. If $\frac{d\lambda }{ds_{2}^{\prime }}\;<\;0$ at this point, it is $s^{}*$.
3. To check for a Type 3 SS, set $s_{2}\;=\;1$ and find $s_{1}$ where $\frac{d\lambda }{ds_{1}^{\prime }}=0$. If $\frac{d\lambda }{ds_{2}^{\prime }}\;>\;0$, that point is $s^{}*$.
4. To check for a Type 4 SS, set $s_{1}\;=\;0$ and find $s_{2}$ where $\frac{d\lambda }{ds_{2}^{\prime }}\;=\;0$. If $\frac{d\lambda }{ds_{1}^{\prime }}\;<\;0$, that point is $s^{}*$.
5. To check for a Type 5 SS, set $s_{1}\;=\;1$ and find $s_{2}$ where $\frac{d\lambda }{ds_{2}^{\prime }}=0$. If $\frac{d\lambda }{ds_{1}^{\prime }}\;>\;0$, that point is $s^{}*$.

These singular strategies $s^{}*$ are potential evolutionary endpoints for **s**. Characterizing their evolutionary and convergence stability can be challenging because **s** is a vector‐valued trait (but see Appendix B). Generating the evolutionary trajectories of **s** using (21), however, may lend insight into general stability patterns.

## Case Studies

We will use this framework to examine two cases where the primary sex ratio depends on maternal condition. Again, the evolving sex ratio phenotype is the vector $s=(s_{1},s_{2})$, and the components of which are the sex ratios used by mothers in each condition.

Our maternal conditions of interest are as follows:

- **Case 1: Maternal age.** Young mothers have sex ratio $s_{1}$, and old mothers have sex ratio $s_{2}$.
- **Case 2: Maternal quality.** Low‐quality mothers have sex ratio $s_{1}$, and high‐quality mothers have sex ratio $s_{2}$.

In both cases, there are two possible conditions for an individual (young and old in Case 1; low‐ and high‐quality in Case 2). Although individuals of different conditions may interbreed (e.g., a high‐quality male may mate with a low‐quality female), a couple's sex ratio is determined by the condition of the female partner (e.g., a couple with a low‐quality female would have sex ratio $s_{1}$ regardless of the male partner's quality).

In each case, we examine the evolutionary trajectories generated by the variance‐constrained, bivariate canonical equation (21), and the types of evolutionarily singular strategies $s^{}*$ (Fig. 3) that result.

Unless otherwise indicated, model parameters are as in Table 1. Our model also makes the following assumptions:

**Table 1:** Two‐sex model parameters. A subscript of *m* indicates male, and a subscript of *f* indicates female. In Case 1, Condition 1 individuals are young and Condition 2 individuals are old. In Case 2, Condition 1 individuals are low quality and Condition 2 individuals are high quality

Parameter	Description	Value
Both Cases		
$C_{m},C_{f}$	Offspring resource costs (resources used per offspring born)	Constants (Case 2) or given by (27) (Case 1b)
$k_{j}$	Reproductive rate (offspring born per time) of Condition *j* mothers	Constants (Case 1a) or given by (26) (Case 1b, Case 2)
$R_{j}$	Resource investment rate (resources put into offspring per time) of Condition *j* mothers	10
$d_{ij}$	Divorce rate of union $u_{ij}$	0.1
$s_{j}$	Primary sex ratio of Condition *j* mothers	Component of **s** (1)
$\mu_{mi}$	Male adult mortality rate in Condition *i*	0.1
$\mu_{fj}$	Female adult mortality rate in Condition *j*	0.1
$U_{m,ij}$	Per capita mating rate of a male in union $u_{ij}$	Given by (14)
$U_{f,ij}$	Per capita mating rate of a female in union $u_{ij}$	Given by (14)
Case 1 (maternal age) only		
$\alpha_{m1},\alpha_{f1}$	Juvenile to young adult maturation rates	0.5
$\alpha_{m2},\alpha_{f2}$	Young adult to old adult maturation rates	0.5
$\mu_{m0},\mu_{f0}$	Juvenile mortality rates	0.1
*β*	Parental mortality intensity factor in (32)	0.2
*I*	Baseline investment rate in (27)	1
Case 2 (maternal quality) only		
$\alpha_{m1},\alpha_{f1}$	Low‐quality juvenile to adult maturation rates	1
$\alpha_{m2},\alpha_{f2}$	High‐quality juvenile to adult maturation rates	1
$\mu_{m,01},\mu_{f,01}$	Low‐quality juvenile mortality rates	0.1
$\mu_{m,02},\mu_{f,02}$	High‐quality juvenile mortality rates	0.1
$c_{i}$	Male competitiveness factor for Condition *i* (40)	$c_{1}=0.1,c_{2}=0.9$
$q_{ij}$	Probability that a Condition *j* female produces Condition *i* offspring, subject to (38)	$q=q_{11}=q_{22}=0.65,q_{21}=q_{12}=1-q$

- A union $u_{ij}$ (Condition *i* male mated with Condition *j* female) has divorce rate $d_{ij}$, reproductive rate $k_{j}$, and primary sex ratio $s_{j}$. Note that the reproductive rate and primary sex ratio are maternally determined.
- Only unions can produce new offspring. Unmated males and females do not reproduce independently.
- Any offspring with a mutant parent also has the mutant phenotype; that is, the mutant genotype is genetically dominant.

Results for all cases are summarized in Table 2.

**Table 2:** Evolutionarily singular strategies $s^{}*$ for primary sex ratios that depend on maternal condition (age or quality)

Case	Offspring cost	Previous predictions	Model results
Maternal age			
Case 1a	Parental mortality (Case 4)^†^	*Young* mothers favor sex inducing less mortality, *old* mothers favor sex inducing more mortality (Charnov 1982)	Results depend on relative reproductive rates of young and old mothers (Figure 6)
Case 1b	Offspring mortality during parental investment (Case 2)^†^	*Young* mothers favor higher mortality sex, *old* mothers favor lower mortality sex (Charlesworth 1977)	Infinitely many selectively neutral sex ratio combinations (Figure 7)
Maternal quality			
Case 2	Offspring resource cost (Case 1)^†^	High‐quality mothers favor the sex with greater variance in reproductive success or value (Trivers and Willard 1973; Leimar 1996)	High‐quality mothers favor the sex with greater variance in reproductive value at *boundary* SS (Table 3)

^†^Corresponding single sex ratio case in Shyu and Caswell (2016)

## Case 1: Maternal Age

Previous studies suggest that sex ratios differ with parental age when male and female offspring are differentially costly. However, different types of offspring costs may result in different bivariate sex ratio patterns.

Differential offspring costs can occur when offspring of one sex induce more parental mortality (Shyu and Caswell 2016, Case 4). Human sons, for instance, reduce maternal longevity more than daughters do (Helle et al. 2002). Younger mothers should thus favor daughters (the less mortality‐inducing sex), while older mothers favor sons (the more mortality‐inducing sex). Charnov (1982) suggested this as an example of senescence through antagonistic pleiotropy (Williams 1957) – that is, genes selected for their beneficial effects early in life (e.g., a lower mortality reproductive strategy) could have negative effects later in life (e.g., a higher mortality reproductive strategy).

Alternatively, differential offspring costs can occur when offspring of a particular sex are more likely to die before independence (Shyu and Caswell 2016, Case 2). In many mammals, also including humans, male offspring have higher *in utero* mortality rates (Trivers 1985; Vatten and Skjaerven 2004; though see also Orzack et al. 2015). Because older mothers are more likely to die or become sterile before they are able to replace lost sons, younger mothers should favor sons (the more mortality‐prone sex), while older mothers favor daughters (the less mortality‐prone sex) (Charlesworth 1977).

These two examples predict opposite trends for human sex ratios with maternal age. Empirical studies have alternatingly found sex ratios to increase (Takahashi 1954), decrease (Pollard 1969; James and Rostron 1985), or be uncorrelated with maternal age (Almagor et al. 1998; Jacobsen et al. 1999). These mixed results may suggest that the effects of various offspring costs vary or even counterbalance in different populations, or that there are additional factors at play.

We will examine how the sex ratios of younger and older mothers are affected by sex‐biased offspring costs. We consider both parental mortality and offspring mortality costs, in turn, through the following two subcases:

- **Case 1a.** Male and female offspring are differentially costly through their effects on parental mortality, similar to Charnov (1982). We previously described a similar single sex ratio model in Shyu and Caswell 2016, Case 4).
- **Case 1b.** Male and female offspring have different mortality rates before independence (during the period of parental investment), similar to Charlesworth (1977). We previously described a similar single sex ratio model in Shyu and Caswell 2016, Case 2).

### Model

We partition males and females into immature juveniles ($m_{0}$, $f_{0}$), young adults ($m_{1}$, $f_{1}$), and old adults ($m_{2}$, $f_{2}$). Only young and old adults can mate to form reproducing unions, and the four possible union types are as follows:

(23) $$\begin{array}{cc} u_{11} & =\text{union}\;\mathrm{of}\;m_{1}\;\text{and}\;f_{1}\\ u_{21} & =\text{union of}\;m_{2}\;\text{and}\;f_{1}\\ u_{12} & =\text{union of}\;m_{1}\;\text{and}\;f_{2}\\ u_{22} & =\text{union of}\;m_{2}\;\text{and}\;f_{2} \end{array}$$

The population vector (2) has 10 stages total:

(24) $$n\left(t\right)={\left(\begin{array}{cccccccccc} m_{0} & m_{1} & m_{2} & f_{0} & f_{1} & f_{2} & u_{11} & u_{21} & u_{12} & u_{22} \end{array}\right)}^{⊺}$$

We will write a model of the form (4), and the next three sections give the matrices **B**,** U**, and **T** in turn.

#### Births (**B**)

Unions with young adult and old adult females use the sex ratios $s_{1}$ and $s_{2}$, respectively, and have characteristic reproductive rates $k_{1}$ and $k_{2}$, respectively. The birth matrix **B** is thus:

(25) $$B=\left(\begin{array}{cccccccccc} 0 & 0 & 0 & 0 & 0 & 0 & s_{1}k_{1} & s_{1}k_{1} & s_{2}k_{2} & s_{2}k_{2}\\ 0 & 0 & 0 & 0 & 0 & 0 & 0 & 0 & 0 & 0\\ 0 & 0 & 0 & 0 & 0 & 0 & 0 & 0 & 0 & 0\\ 0 & 0 & 0 & 0 & 0 & 0 & \left(1-s_{1}\right)k_{1} & \left(1-s_{1}\right)k_{1} & \left(1-s_{2}\right)k_{2} & \left(1-s_{2}\right)k_{2}\\ 0 & 0 & 0 & 0 & 0 & 0 & 0 & 0 & 0 & 0\\ 0 & 0 & 0 & 0 & 0 & 0 & 0 & 0 & 0 & 0\\ 0 & 0 & 0 & 0 & 0 & 0 & 0 & 0 & 0 & 0\\ 0 & 0 & 0 & 0 & 0 & 0 & 0 & 0 & 0 & 0\\ 0 & 0 & 0 & 0 & 0 & 0 & 0 & 0 & 0 & 0\\ 0 & 0 & 0 & 0 & 0 & 0 & 0 & 0 & 0 & 0 \end{array}\right)$$

In Case 1a, where male and female offspring have different effects on parental mortality, the $k_{j}$ are fixed rates. In Case 1b, where male and female offspring have different mortality rates during the period of parental investment, $k_{j}$ becomes:

(26) $$k_{j}=\frac{R_{j}}{s_{j}C_{m}+\left(1-s_{j}\right)C_{f}}$$

where $R_{j}$ is the mother's rate of resource investment (total resources put into offspring per time), and $C_{m}$ and $C_{f}$ are the average male and female offspring resource costs (resources consumed per offspring born). In Shyu and Caswell 2016, Case 2), these costs are shown to be:

(27) $$\begin{array}{cc} C_{m} & =\frac{I}{\mu_{m0}}\left(1-e^{\frac{-\mu_{m0}}{\alpha_{m1}}}\right)\\ C_{f} & =\frac{I}{\mu_{f0}}\left(1-e^{\frac{-\mu_{f0}}{\alpha_{f1}}}\right) \end{array}$$

where *I* is a constant baseline investment rate, $\alpha_{m1}$ and $\alpha_{f1}$ are the male and female juvenile to adult maturation rates, and $\mu_{m0}$ and $\mu_{f0}$ are the male and female juvenile mortality rates.

#### Union formation (**U**)

The union formation matrix **U** contains per capita mating rates of each union type. Using a harmonic mean mating function as in (13), the per capita mating functions (14) are:

(28) $$\begin{array}{cc} U_{m11} & =U_{m21}=\frac{2f_{1}}{m+f}\\ U_{m12} & =U_{m22}=\frac{2f_{2}}{m+f}\\ U_{f11} & =U_{f12}=\frac{2m_{1}}{m+f}\\ U_{f21} & =U_{f22}=\frac{2m_{2}}{m+f} \end{array}$$

where $m=m_{1}+m_{2}$ and $f=f_{1}+f_{2}$.

The matrix **U** is then:

(29) $$U=\left(\begin{array}{cccccccccc} 0 & 0 & 0 & 0 & 0 & 0 & 0 & 0 & 0 & 0\\ 0 & -(U_{m11}+U_{m12}) & 0 & 0 & 0 & 0 & 0 & 0 & 0 & 0\\ 0 & 0 & -(U_{m21}+U_{m22}) & 0 & 0 & 0 & 0 & 0 & 0 & 0\\ 0 & 0 & 0 & 0 & 0 & 0 & 0 & 0 & 0 & 0\\ 0 & 0 & 0 & 0 & -(U_{f11}+U_{f21}) & 0 & 0 & 0 & 0 & 0\\ 0 & 0 & 0 & 0 & 0 & -(U_{f12}+U_{f22}) & 0 & 0 & 0 & 0\\ 0 & \frac{1}{2}U_{m11} & 0 & 0 & \frac{1}{2}U_{f11} & 0 & 0 & 0 & 0 & 0\\ 0 & 0 & \frac{1}{2}U_{m21} & 0 & \frac{1}{2}U_{f21} & 0 & 0 & 0 & 0 & 0\\ 0 & \frac{1}{2}U_{m12} & 0 & 0 & 0 & \frac{1}{2}U_{f12} & 0 & 0 & 0 & 0\\ 0 & 0 & \frac{1}{2}U_{m22} & 0 & 0 & \frac{1}{2}U_{f22} & 0 & 0 & 0 & 0 \end{array}\right)$$

#### Transitions (**T**)

Each stage has a characteristic mortality rate:

(30) $$\mu_{xs}\;\text{where}\;x\in \left\{m,f\right\}\;\text{and}\;s\in \left\{0,1,2\right\}$$

If offspring impose parental mortality (Case 1a), the mortality rates of individuals in reproducing unions is greater than that of unmated individuals. Let $\mu_{xs}$ (30) be the mortality rate of an unmated individual, and $\mu_{xs}^{ij}$ be the mortality rate of a mated individual in union $u_{ij}$. Similar to Shyu and Caswell 2016, Case 4), $\mu_{xs}^{ij}$ is increased from $\mu_{xs}$ by an amount $\gamma_{j}$:

(31) $$\mu_{xs}^{ij}=\mu_{xs}+\gamma_{j}$$

Let *β* be a nonnegative constant that modulates the intensity of offspring‐induced mortality. In Case 1a, *β* is a positive constant. In Case 1b, offspring do not affect parental mortality, so *β* is 0. Then $\gamma_{j}$ can be written as:

(32) $$\begin{array}{cc} \gamma_{1} & =\beta k_{1}\left[s_{1}C_{m}+\left(1-s_{1}\right)C_{f}\right]\\ \gamma_{2} & =\beta k_{2}\left[s_{2}C_{m}+\left(1-s_{2}\right)C_{f}\right] \end{array}$$

Again, the average offspring costs $C_{m}$ and $C_{f}$ are given by (27).

Juveniles mature into young adults at a rate $\alpha_{m1}$ for males and $\alpha_{f1}$ for females, young adults mature into old adults at a rate $\alpha_{m2}$ for males and $\alpha_{f2}$ for females, and old adults cannot transition into any other prior stage. It is possible for a couple of one type to transition into another if a partner matures (e.g., a $u_{11}$ union will become a $u_{12}$ union if the young female partner matures into an old female). Unions may also divorce at a rate $d_{ij}$ or dissolve due to partner death, with mortality rates given by (31). The full transition matrix is $T=[T_{1}|T_{2}]$ where:

(33) $$\begin{array}{cc} T_{1} & =\left(\begin{array}{cccccc} -(\mu_{m0}+\alpha_{m1}) & 0 & 0 & 0 & 0 & 0\\ \alpha_{m1} & -(\mu_{m1}+\alpha_{m2}) & 0 & 0 & 0 & 0\\ 0 & \alpha_{m2} & -\mu_{m2} & 0 & 0 & 0\\ 0 & 0 & 0 & -(\mu_{f0}+\alpha_{f1}) & 0 & 0\\ 0 & 0 & 0 & \alpha_{f1} & -(\mu_{f1}+\alpha_{f2}) & 0\\ 0 & 0 & 0 & 0 & \alpha_{f2} & -\mu_{f2}\\ 0 & 0 & 0 & 0 & 0 & 0\\ 0 & 0 & 0 & 0 & 0 & 0\\ 0 & 0 & 0 & 0 & 0 & 0\\ 0 & 0 & 0 & 0 & 0 & 0 \end{array}\right)\\ T_{2} & =\left(\begin{array}{cccc} 0 & 0 & 0 & 0\\ \left(\mu_{f1}+\gamma_{11}+d_{11}\right) & 0 & \left(\mu_{f2}+\gamma_{12}+d_{12}\right) & 0\\ 0 & \left(\mu_{f1}+\gamma_{21}+d_{21}\right) & 0 & \left(\mu_{f2}+\gamma_{22}+d_{22}\right)\\ 0 & 0 & 0 & 0\\ \left(\mu_{m1}+\gamma_{11}+d_{11}\right) & \left(\mu_{m2}+\gamma_{21}+d_{21}\right) & 0 & 0\\ 0 & 0 & \left(\mu_{m1}+\gamma_{12}+d_{12}\right) & \left(\mu_{m2}+\gamma_{22}+d_{22}\right)\\ -(\mu_{m1}+\mu_{f1}+2\gamma_{11}+\\ d_{11}+\alpha_{m2}+\alpha_{f2}) & 0 & 0 & 0\\ \alpha_{m2} & -(\mu_{m2}+\mu_{f1}+2\gamma_{21}+d_{21}+\alpha_{f2}) & 0 & 0\\ \alpha_{f2} & 0 & -(\mu_{m1}+\mu_{f2}+2\gamma_{12}+d_{12}+\alpha_{m2}) & 0\\ 0 & \alpha_{f2} & \alpha_{m2} & -(\mu_{m2}+\mu_{f2}+2\gamma_{22}+d_{22}) \end{array}\right) \end{array}$$

### Results (Case 1a: Parental mortality cost)

**Figure 4:**
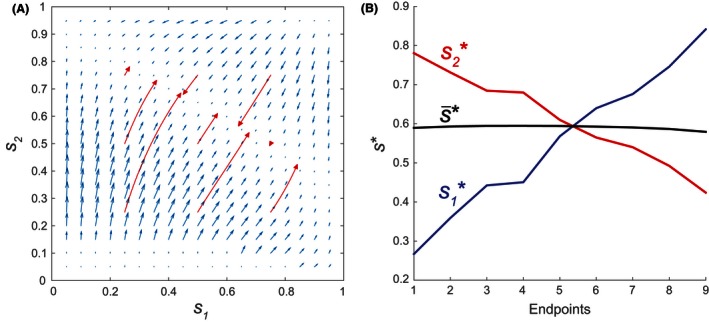
Evolutionary trajectories for Case 1a when all mothers have the same vital and reproductive rates ($k_{1}\;=\;k_{2}\;=\;10$). Offspring costs $C_{m}\;=\;0.4,C_{f}\;=\;0.6$; other model parameters are as in Table 1. (A) Selection gradients as functions of the age‐specific sex ratios $s_{1}$ (younger females) and $s_{2}$ (older females). Blue arrows indicate the directions and relative magnitudes of the selection gradient (16). Red arrows indicate the evolutionary trajectories of **s** given by the canonical equation (21). (B) The young, old, and average primary sex ratios $s_{1}$, $s_{2}$, and $\overset{¯}{s}$ (34) at the trajectory endpoints in Fig. 4A.

Suppose that male and female offspring have different costs on parental mortality. As an example, let female offspring be somewhat more costly [$C_{f}\;=\;0.6,C_{m}\;=\;0.4$ in (32)]. As a baseline case, consider the scenario where young and old mothers have the same reproductive rate $k_{1}\;=\;k_{2}$. In this case, all mothers have the same vital rates and reproductive abilities, regardless of their age. Figure 4A shows the direction and relative magnitudes of the selection gradients (blue), as functions of the age‐specific sex ratios $s_{1}$ and $s_{2}$.

Note that the evolutionary trajectories of **s** (red) converge not to one singular strategy, but instead to a whole line of strategies. Changing the offspring costs $C_{m}$ and $C_{f}$ yields qualitatively similar behavior (results not shown). Along this line of strategies, both components of the selection gradient are 0, indicating the absence of selective pressure. Thus, if **s** is initialized at different values of $s_{1}$ and $s_{2}$, its corresponding evolutionary endpoints may differ significantly. However, the same average primary sex ratio:

(34) $$\overset{¯}{s}=\frac{s_{1}\left(u_{11}+u_{21}\right)+s_{2}\left(u_{12}+u_{22}\right)}{\sum u_{ij}}$$

is shared by all the trajectory endpoints, where $\overset{¯}{s}\;\approx \;0.6$ (Fig. 4B). This is the same value expected from the equal investment principle in the single sex ratio case (Shyu and Caswell 2016), where the optimal single sex ratio $s^{}*$ evolves to:

(35) $$s^{}*=\frac{C_{f}}{C_{m}+C_{f}}$$

Ultimately, it appears that any combination of $s_{1}$ and $s_{2}$ that leads to $\overset{¯}{s}\;\approx \;0.6$ is a selectively neutral point on a line of singular strategies. Presumably because young and old females have similar reproductive rates, male and female offspring production can be partitioned between them in an infinite number of ways.

**Figure 5:**
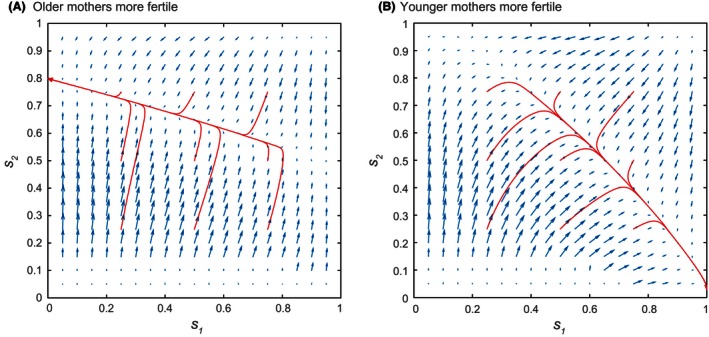
Evolutionary trajectories for Case 1a when young and old mothers have different reproductive rates. Offspring costs $C_{m}\;=\;0.4,C_{f}\;=\;0.6$; other model parameters are as in Table 1. (A) Trajectories when older mothers are more fertile ($k_{1}\;=\;5,k_{2}\;=\;15$). (B) Trajectories when younger mothers are more fertile ($k_{1}\;=\;15,k_{2}\;=\;5$).

Now consider the case where the reproductive rate $k_{j}$ changes with age. When younger and older mothers are sufficiently different, the line of selectively neutral strategies disappears, and **s** converges to a single endpoint $s^{}*$ regardless of its initial condition (Fig. 5). The methods in the section “Equilibrium evolutionary dynamics” identify these endpoints as boundary SSs. If the reproductive rate increases with age ($k_{1}<k_{2}$, Fig. 5A), $s_{1}$ evolves to 0 (Type 4 SS), meaning that younger mothers are producing only the more costly females. If the reproductive rate decreases with age ($k_{1}>k_{2}$, Fig. 5B), $s_{1}$ evolves to 1 (Type 5 SS), so that younger mothers are producing only the less costly males.

**Figure 6:**
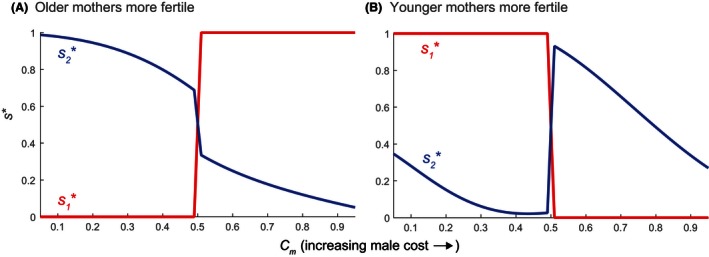
Sex ratio singular strategies $s^{}*$ ($s_{1}^{}*$ for younger females, $s_{2}^{}*$ for older females) as a function of the male offspring cost $C_{m}$, which affects parental mortality via (32). The female offspring cost $C_{f}\;=\;1-C_{m}$ (more costly males mean less costly females); other model parameters are as given in Table 1. (A) Values of $s^{}*$ when older mothers are more fertile ($k_{1}\;=\;5,k_{2}\;=\;15$). (B) Values of $s^{}*$ when younger mothers are more fertile ($k_{1}\;=\;15,k_{2}\;=\;5$).

Figure 6 shows $s^{}*$ for a range of offspring costs on parental mortality. If the reproductive rate increases with age (Fig. 6A), older females avoid the costly sex, while younger females compensate by producing only the costly sex. When $C_{m}\;<\;C_{f}$, for example, young mothers produce only the more costly females ($s_{1}\;=\;0$); when $C_{m}\;>\;C_{f}$, they switch to producing only the more costly males ($s_{1}\;=\;1$). The older sex ratio $s_{2}$ favors the less mortality‐inducing sex but, unlike $s_{1}$, never evolves to exclusively producing a single sex. When costs become increasingly unequal ($C_{m}\;\gg \;C_{f}$ or $C_{m}\;\ll \;C_{f}$), $s_{2}$ diverges more from $s_{1}$.

If the reproductive rate decreases with age (Fig. 6B), the directions of the sex ratio biases reverse. Younger females produce only the cheaper sex ($s_{1}\;=\;0$ or $s_{1}\;=\;1$), forcing older females to produce the costlier sex. When costs become increasingly unequal ($C_{m}\to 0$ or $C_{m}\to 1$), we see that $s_{2}$ diverges less from $s_{1}$. Older mothers can produce more of the costlier sex when the sex‐specific costs are similar ($C_{m}\approx C_{f}$), but less when cost differences are high ($C_{m}\;\gg \;C_{f}$ or $C_{m}\;\ll \;C_{f}$). This contrasts with how younger females produce only the costlier sex (Fig. 6A)

### Results (Case 1b: Parental resource cost)

**Figure 7:**
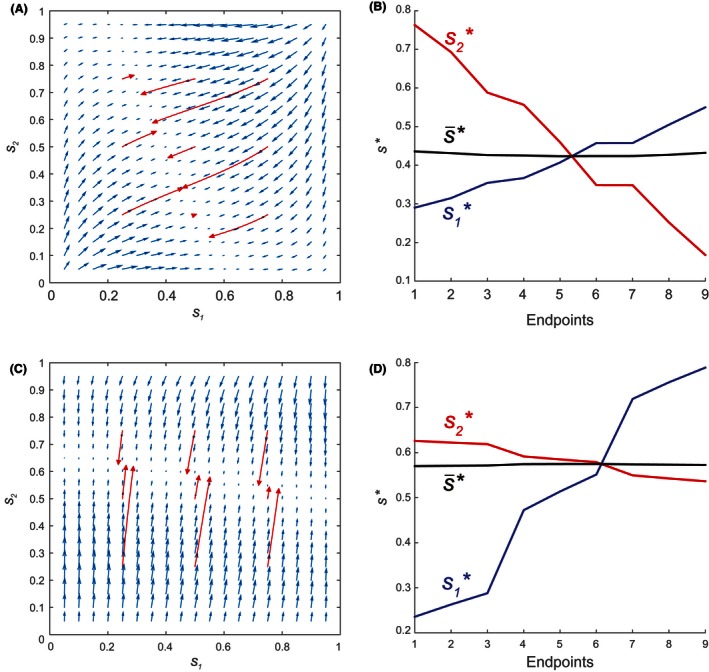
Example evolutionary trajectories and the sex ratios ($s_{1}$ for younger females, $s_{2}$ for older females) at their endpoints for Case 1b. (A) Trajectories and (B) endpoints for $C_{m}\;=\;0.2,C_{f}\;=\;0.8,\mu_{f1}\;=\;0.1,\mu_{f2}\;=\;0.5,k_{1}\;=\;15,k_{2}\;=\;5$. (C) Trajectories and (D) endpoints for $C_{m}\;=\;0.8,C_{f}\;=\;0.2,\mu_{f1}\;=\;0.5,\mu_{f2}\;=\;0.1,k_{1}\;=\;5,k_{2}\;=\;15$. Other model parameters are as given in Table 1.

Suppose that male and female offspring do not affect parental mortality, but experience different mortality rates during the period of parental investment. As in Case 1a with identical mothers (Fig. 4), **s** ultimately converges to a selectively neutral line of singular strategies (Fig. 7). Unlike Case 1a, this line persists even when young and old females differ in reproductive rates $k_{j}$ or baseline mortality rates $\mu_{fj}$. Once again, all combinations of $s_{1}$ and $s_{2}$ on the line share a similar average primary sex ratio $\overset{¯}{s}$ (Fig. 7B and 7D).

As a result, the population may converge to any one of an infinite number of sex ratio combinations, which are selectively neutral and have same average primary sex ratio. The sex ratios observed in the long‐term may accordingly vary with the initial state of **s**.

## Case 2: Maternal Quality

As described in the introduction, the Trivers‐Willard hypothesis (Trivers and Willard 1973) predicts that the primary sex ratio produced by a mother should depend on maternal quality. Specifically, high‐quality females will preferentially invest in the sex whose reproductive success varies most with quality. This hypothesis has three main assumptions:

1. An offspring's quality carries into adulthood. In comparison with their low‐quality counterparts, high‐quality offspring will be larger, stronger, or have higher social ranks throughout their lifetimes. These advantages ultimately confer greater reproductive success or higher reproductive value (Leimar 1996). We will specifically consider two main advantages that high‐quality adults have over low‐quality adults. These advantages concern the male competitive factor $c_{i}$ and female resource investment rate $R_{j}$ described in the subsequent sections “Union formation (**U**)” and “Births (**B**)”, respectively. We will assume that one or both of the following advantages is present.

- High‐quality males obtain a greater proportion of total matings, and thus have a greater competiveness factor ($c_{2}\;>\;c_{1}$).
- High‐quality females invest more resources into offspring production, and thus have a greater resource investment rate ($R_{2}\;>\;R_{1}$).

The quality of an offspring is correlated with the quality of its parent (usually the mother). As shown in the subsequent section “Births (**B**)”, we incorporate maternal quality transmission via a quality inheritance probability $q_{ij}$. High‐quality females will be more likely to produce high‐quality offspring, while low‐quality females will be more likely to produce low‐quality offspring. Maternal transmission of quality occurs in many species, especially those with small broods (Trivers and Willard 1973); high‐ranking red deer mothers, for instance, produce larger and more dominant offspring (Clutton‐Brock et al. 1986). Quality transmission also affects the value of female offspring; when offspring quality depends mostly on maternal quality, high‐quality females are more productive in the long run (Leimar 1996).
One sex (usually males) has a greater variance in reproductive success with quality. Though the reproductive potential of both males and females may vary with quality, one sex varies more than the other, depending on the relative advantages of high‐quality males and females. Although reproductive success is often framed in terms of number of offspring, Leimar (1996) showed that reproductive values are more relevant for sex ratio evolution. As described in the introduction, we express the notion of “variance in reproductive success” in terms of male and female reproductive value ratios. In polygynous ungulates, for example, males have the greater reproductive variance. Dominant high‐quality males monopolize breeding opportunities and have many more offspring than low‐quality males, while high‐quality females have only moderately more offspring than low‐quality females (Trivers and Willard 1973). In other species, females have the greater reproductive variance. Female baboons and macaques, for example, are more strongly affected by maternal quality due to their inheritance of maternal rank. As a result, the sex ratios of high‐ranking mothers are biased toward female offspring (Silk 1983).

### Model

Trivers and Willard based their analysis on a verbal argument that implicitly relies on the principle of equal investment. Here, we explore similar questions in a structured model that includes multiple stages, qualities, and pair formation.

The population in our model consists of male and female low‐quality juveniles ($m_{01}$, $f_{01}$), high‐quality juveniles ($m_{02}$, $f_{02}$), low‐quality adults ($m_{1}$, $f_{1}$), and high‐quality adults ($m_{2}$, $f_{2}$). Low and high‐quality adults interbreed to form four types of unions, as in (23).

The population vector (2) has 12 stages total:

(36) $$n\left(t\right)={\left(\begin{array}{cccccccccccc} m_{01} & m_{02} & m_{1} & m_{2} & f_{01} & f_{02} & f_{1} & f_{2} & u_{11} & u_{21} & u_{12} & u_{22} \end{array}\right)}^{⊺}$$

Again, we will write a model of the form (4), and the next three sections give the matrices **B**,** U**, and **T** in turn.

#### Births (**B**)

Unlike Case 1, offspring do not have different mortality rates or impose parental mortality. However, the production of male and female offspring requires different amounts of resources, as in Shyu and Caswell 2016, Case 1). Producing a male offspring costs $C_{m}$ units of resources per time, while a female offspring costs $C_{f}$ units of resources per time. Each union's total rate of resource investment in offspring production is determined by maternal quality, where:

(37) $$\begin{array}{cc} R_{1} & =\text{rate of resource investment by low‐quality females}\\ R_{2} & =\text{rate of resource investment by high‐quality females} \end{array}$$

Because high‐quality females have more resources for producing offspring, $R_{2}\;>\;R_{1}$. The corresponding low‐ and high‐quality female reproductive rates, $k_{1}\left[R_{1}\right]$ and $k_{2}\left[R_{2}\right]$, are given by (26).

Let $q_{ij}$ be the probability that a female of quality *j* produces quality *i* offspring. We assume inheritance of quality, in that mothers are equally or more likely to produce offspring of the same quality. Thus, the $q_{ij}$ must satisfy the following conditions:

(38) $$\begin{array}{cc} q_{11} & +q_{21}=1\\ q_{12} & +q_{22}=1\\ q_{22} & >q_{12}\to q_{22}\geq 0.5\\ q_{11} & >q_{21}\to q_{11}\geq 0.5 \end{array}$$

The complete birth matrix **B** is:

(39) $$B=\left(\begin{array}{cccccccccccc} 0 & 0 & 0 & 0 & 0 & 0 & 0 & 0 & s_{1}k_{1}\left[R_{1}\right]q_{11} & s_{1}k_{1}\left[R_{1}\right]q_{11} & s_{2}k_{2}\left[R_{2}\right]q_{12} & s_{2}k_{2}\left[R_{2}\right]q_{12}\\ 0 & 0 & 0 & 0 & 0 & 0 & 0 & 0 & s_{1}k_{1}\left[R_{1}\right]q_{21} & s_{1}k_{1}\left[R_{1}\right]q_{21} & s_{2}k_{2}\left[R_{2}\right]q_{22} & s_{2}k_{2}\left[R_{2}\right]q_{22}\\ 0 & 0 & 0 & 0 & 0 & 0 & 0 & 0 & 0 & 0 & 0 & 0\\ 0 & 0 & 0 & 0 & 0 & 0 & 0 & 0 & 0 & 0 & 0 & 0\\ 0 & 0 & 0 & 0 & 0 & 0 & 0 & 0 & \left(1-s_{1}\right)k_{1}\left[R_{1}\right]q_{11} & \left(1-s_{1}\right)k_{1}\left[R_{1}\right]q_{11} & \left(1-s_{2}\right)k_{2}\left[R_{2}\right]q_{12} & \left(1-s_{2}\right)k_{2}\left[R_{2}\right]q_{12}\\ 0 & 0 & 0 & 0 & 0 & 0 & 0 & 0 & \left(1-s_{1}\right)k_{1}\left[R_{1}\right]q_{21} & \left(1-s_{1}\right)k_{1}\left[R_{1}\right]q_{21} & \left(1-s_{2}\right)k_{2}\left[R_{2}\right]q_{22} & \left(1-s_{2}\right)k_{2}\left[R_{2}\right]q_{22}\\ 0 & 0 & 0 & 0 & 0 & 0 & 0 & 0 & 0 & 0 & 0 & 0\\ 0 & 0 & 0 & 0 & 0 & 0 & 0 & 0 & 0 & 0 & 0 & 0\\ 0 & 0 & 0 & 0 & 0 & 0 & 0 & 0 & 0 & 0 & 0 & 0\\ 0 & 0 & 0 & 0 & 0 & 0 & 0 & 0 & 0 & 0 & 0 & 0\\ 0 & 0 & 0 & 0 & 0 & 0 & 0 & 0 & 0 & 0 & 0 & 0\\ 0 & 0 & 0 & 0 & 0 & 0 & 0 & 0 & 0 & 0 & 0 & 0 \end{array}\right)$$

#### Union formation (**U**)

Each union type $u_{ij}$ is formed at a mating rate $U_{ij}$ determined by the mating preference functions described in the earlier section “Mating preferences”. Assume that males are not picky in their choice of females, so that the male preference distribution is given by the random mating preference (10). However, as per our first assumption, females may prefer to mate with high‐quality males. Thus, the female preference distribution will be given by the biased mating preference (11).

Low and high‐quality males have competitiveness factors $c_{1}$ and $c_{2}$, respectively. Since high‐quality males are more likely to obtain mates than their low‐quality counterparts, $c_{2}\;>\;c_{1}$. Because $c_{1}\;+\;c_{2}\;=\;1$ in accordance with (8):

(40) $$c_{2}>c_{1}\to c_{1}<0.5$$

Using the harmonic mean mating function (13), the per capita mating functions (14) become:

(41) $$\begin{array}{cc} U_{m11}=\frac{2c_{1}f_{1}}{c_{1}\left(f+m_{1}\right)+c_{2}m_{2}} & U_{f11}=\frac{2c_{1}m_{1}}{c_{1}\left(f+m_{1}\right)+c_{2}m_{2}}\\ U_{m21}=\frac{2c_{2}f_{1}}{c_{1}m_{1}+c_{2}\left(f+m_{2}\right)} & U_{f21}=\frac{2c_{2}m_{2}}{c_{1}m_{1}+c_{2}\left(f+m_{2}\right)}\\ U_{m12}=\frac{2c_{1}f_{2}}{c_{1}\left(f+m_{1}\right)+c_{2}m_{2}} & U_{f12}=\frac{2c_{1}m_{1}}{c_{1}\left(f+m_{1}\right)+c_{2}m_{2}}\\ U_{m22}=\frac{2c_{2}f_{2}}{c_{1}m_{1}+c_{2}\left(f+m_{2}\right)} & U_{f22}=\frac{2c_{2}m_{2}}{c_{1}m_{1}+c_{2}\left(f+m_{2}\right)} \end{array}$$

where $m\;=\;m_{1}\;+\;m_{2}$ and $f\;=\;f_{1}\;+\;f_{2}$.

The union matrix **U** is:

(42) $$U=\left(\begin{array}{cccccccccccc} 0 & 0 & 0 & 0 & 0 & 0 & 0 & 0 & 0 & 0 & 0 & 0\\ 0 & 0 & 0 & 0 & 0 & 0 & 0 & 0 & 0 & 0 & 0 & 0\\ 0 & 0 & -(U_{m11}+U_{m12}) & 0 & 0 & 0 & 0 & 0 & 0 & 0 & 0 & 0\\ 0 & 0 & 0 & -(U_{m21}+U_{m22}) & 0 & 0 & 0 & 0 & 0 & 0 & 0 & 0\\ 0 & 0 & 0 & 0 & 0 & 0 & 0 & 0 & 0 & 0 & 0 & 0\\ 0 & 0 & 0 & 0 & 0 & 0 & 0 & 0 & 0 & 0 & 0 & 0\\ 0 & 0 & 0 & 0 & 0 & 0 & -(U_{f11}+U_{f21}) & 0 & 0 & 0 & 0 & 0\\ 0 & 0 & 0 & 0 & 0 & 0 & 0 & -(U_{f12}+U_{f22}) & 0 & 0 & 0 & 0\\ 0 & 0 & \frac{1}{2}U_{m11} & 0 & 0 & 0 & \frac{1}{2}U_{f11} & 0 & 0 & 0 & 0 & 0\\ 0 & 0 & 0 & \frac{1}{2}U_{m21} & 0 & 0 & \frac{1}{2}U_{f21} & 0 & 0 & 0 & 0 & 0\\ 0 & 0 & \frac{1}{2}U_{m12} & 0 & 0 & 0 & 0 & \frac{1}{2}U_{f12} & 0 & 0 & 0 & 0\\ 0 & 0 & 0 & \frac{1}{2}U_{m22} & 0 & 0 & 0 & \frac{1}{2}U_{f22} & 0 & 0 & 0 & 0 \end{array}\right)$$

#### Transitions (**T**)

Mortality rates are the same for individuals of the same sex and quality, regardless of whether they are in unions. Again, unions dissolve due to divorce rates $d_{ij}$ or partner deaths (with mortality rates $\mu_{m1}$, $\mu_{f1}$, $\mu_{m2}$, and $\mu_{f2}$).

Low‐quality juveniles mature into low‐quality adults at a rate $\alpha_{m1}$ for males and $\alpha_{f1}$ for females. High‐quality juveniles mature into high‐quality adults at a rate $\alpha_{m2}$ for males and $\alpha_{f2}$ for females. Individuals cannot transition between different qualities.

The transition matrix is $T=[T_{1}|T_{2}]$ where:

(43) $$\begin{array}{cc} T_{1} & =\left(\begin{array}{cccccccc} -(\mu_{m,01}+\alpha_{m1}) & 0 & 0 & 0 & 0 & 0 & 0 & 0\\ 0 & -(\mu_{m,02}+\alpha_{m2}) & 0 & 0 & 0 & 0 & 0 & 0\\ \alpha_{m1} & 0 & -\mu_{m1} & 0 & 0 & 0 & 0 & 0\\ 0 & \alpha_{m2} & 0 & -\mu_{m2} & 0 & 0 & 0 & 0\\ 0 & 0 & 0 & 0 & -(\mu_{f,01}+\alpha_{f1}) & 0 & 0 & 0\\ 0 & 0 & 0 & 0 & 0 & -(\mu_{f,02}+\alpha_{f2}) & 0 & 0\\ 0 & 0 & 0 & 0 & \alpha_{f1} & 0 & -\mu_{f1} & 0\\ 0 & 0 & 0 & 0 & 0 & \alpha_{f2} & 0 & -\mu_{f2}\\ 0 & 0 & 0 & 0 & 0 & 0 & 0 & 0\\ 0 & 0 & 0 & 0 & 0 & 0 & 0 & 0\\ 0 & 0 & 0 & 0 & 0 & 0 & 0 & 0\\ 0 & 0 & 0 & 0 & 0 & 0 & 0 & 0 \end{array}\right)\\ T_{2} & =\left(\begin{array}{cccc} 0 & 0 & 0 & 0\\ 0 & 0 & 0 & 0\\ \left(\mu_{f1}+d_{11}\right) & 0 & \left(\mu_{f2}+d_{12}\right) & 0\\ 0 & \left(\mu_{f1}+d_{21}\right) & 0 & \left(\mu_{f2}+d_{22}\right)\\ 0 & 0 & 0 & 0\\ 0 & 0 & 0 & 0\\ \left(\mu_{m1}+d_{11}\right) & \left(\mu_{m2}+d_{21}\right) & 0 & 0\\ 0 & 0 & \left(\mu_{m1}+d_{12}\right) & \left(\mu_{m2}+d_{22}\right)\\ -(\mu_{m1}+\mu_{f1}+d_{11}) & 0 & 0 & 0\\ 0 & -(\mu_{m2}+\mu_{f1}+d_{21}) & 0 & 0\\ 0 & 0 & -(\mu_{m1}+\mu_{f2}+d_{12}) & 0\\ 0 & 0 & 0 & -(\mu_{m2}+\mu_{f2}+d_{22}) \end{array}\right) \end{array}$$

### Calculating variance in reproductive success

Recall that the Trivers‐Willard hypothesis requires individuals of different qualities (here, high and low quality), a correlation between parent and offspring quality (*q* ≥ 0.5), and that one sex has a greater variance (larger differential) in reproductive success with quality.

The hypothesis predicts that high‐quality females preferentially invest in the sex with the greater variance in reproductive success (Trivers and Willard 1973). Testing this hypothesis requires two quantities: a measure of reproductive success for each sex, and a measure of the “variance” in reproductive success (i.e., how much reproductive success varies between high and low‐quality individuals of a given sex).

#### Reproductive success

Though reproductive success is sometimes measured as number of offspring (Clutton‐Brock et al. 1984, 1986), Leimar (1996) showed that reproductive value (the present value of all future offspring) was a more relevant index of reproductive success. This is especially true when the probability of maternal quality transmission is high; if females are more likely than males to pass their quality to offspring, a high‐quality female may still have high reproductive success, in that her reproductive value is large even if her number of offspring is not (West 2009).

Here, we use the demographic definition of reproductive value, which depends on the matrix model. Specifically, the dominant left eigenvector **v** of the projection matrix $A(\hat{p})$ is a vector of stage‐specific reproductive values (shown in age‐structured models by Goodman 1968; extended to stage‐structured models by Caswell and Werner 1978 and others). Entry *i* of **v** corresponds to the reproductive value $v_{i}$ of stage *i*.

Although different stages of a given sex will have different reproductive values, the *juvenile* (newborn) reproductive values should be the most relevant indices of reproductive success for each sex (Appendix A).

#### Variance in reproductive success

The “variance” in reproductive success can be written as the juvenile male and female reproductive value ratios (RVRs) at equilibrium (Leimar 1996). Note that reproductive values are defined only up to a multiplicative constant, so taking the ratios of reproductive values removes this constant factor.

Define the male reproductive value ratio *MRVR* as:

(44) $$MRVR=\frac{v_{m,02}}{v_{m,01}}$$

where $v_{m,02}$ and $v_{m,01}$ are the reproductive values of high and low‐quality juvenile males, respectively.

Similarly, the female reproductive value ratio *FRVR* is:

(45) $$FRVR=\frac{v_{f,02}}{v_{f,01}}$$

where $v_{f,02}$ and $v_{f,01}$ are the reproductive values of high‐ and low‐quality juvenile females, respectively.

We will use *MRVR* and *FRVR* to represent the variance in reproductive success (between high‐ and low‐quality individuals) for males and females, respectively.

#### Predictions of the Trivers‐Willard hypothesis

When *MRVR* > *FRVR* (males have greater variance in reproductive success than females), high‐quality mothers should be more likely than low‐quality mothers to produce sons. As a result, we would expect that $s_{2}\;>\;s_{1}$. Given the biological constraints (19), one of the following two cases should thus occur (Leimar 1996).

(46) $$\begin{array}{cc} & s_{1}=0\;\;\mathrm{and}\;\;0<s_{2}<1\\ & 0<s_{1}<1\;\;\mathrm{and}\;\;s_{2}=1 \end{array}$$

When *FRVR* > *MRVR* (females have greater variance in reproductive success than males), high‐quality mothers should be more likely than low‐quality mothers to produce daughters. As a result, we would expect that $s_{2}\;<\;s_{1}$, and that one of the following two cases should occur (Leimar 1996).

(47) $$\begin{array}{cc} & s_{1}=1\;\;\mathrm{and}\;\;0<s_{2}<1\\ & 0<s_{1}<1\;\;\mathrm{and}\;\;s_{2}=0 \end{array}$$

### Results

As described at the beginning of “Case 2: Maternal quality”, we consider two advantages that high‐quality individuals may have over low‐quality individuals. High‐quality males may be more attractive and competitive mates than low‐quality males ($c_{2}\;>\;c_{1}$), which affects the male variance in reproductive success. Alternatively, high‐quality females may be more productive and have a greater resource investment rate than low‐quality females ($R_{2}\;>\;R_{1}$), which affects the female variance in reproductive success. We will determine how $s^{}*$ evolves in both cases.

#### Male variance in reproductive success

Male variance in reproductive success depends on the difference between the low‐quality male competitiveness factor $c_{1}$ from (40) and the high‐quality male competitiveness factor $c_{2}\;=\;1-c_{1}$. Because high‐quality males obtain more matings, $c_{2}\;>\;c_{1}$. As $c_{1}$ increases ($c_{2}$ decreases), the proportion of females mating with a low‐quality male (not mating with a high‐quality male) also increases.

**Figure 8:**
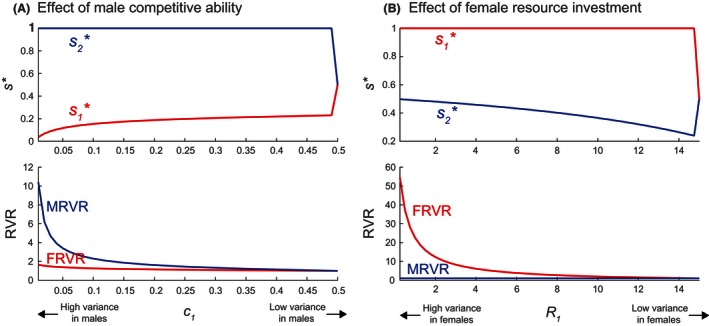
Singular strategies $s^{}*$ ($s_{1}^{}*$ for low‐quality females, $s_{2}^{}*$ for high‐quality females) and reproductive value ratios *MRVR*,* FRVR* as functions of (A) the low‐quality male competitiveness factor $c_{1}$, where the high‐quality male competitiveness factor is $c_{2}\;=\;1-c_{1}$ (with $R_{1}\;=\;R_{2}\;=\;15$), and (B) the low‐quality female investment rate $R_{1}$, where the high‐quality female resource investment rate $R2\;=\;30-R_{1}$ (with $c_{1}\;=\;c_{2}\;=\;0.5$). Other model parameters are as given in Table 1.

Figure 8A shows how *MRVR*,* FRVR*, and $s^{}*$ vary with $c_{1}$. On the left side of the *x*‐axis, males have high variance in competitive ability ($c_{1}\;=\;0.01,c_{2}\;=\;0.99$); on the right side of the *x*‐axis, males have no variance in competitive ability ($c_{1}\;=\;c_{2}\;=\;0.5$). As a result, the variance in male reproductive success, as given by *MRVR*, is highest on the left and converges to 1 on the right.

We have assumed that high and low‐quality females are equally productive ($R_{2}\;=\;R_{1}$), so that there is almost no variance in female reproductive success (*FRVR* ≈ 1). As a result, *MRVR* ≥ *FRVR* at all $s^{}*$. However, note that, at low $c_{1}$, *FRVR* is slightly >1, indicating that high‐quality females are somewhat more successful than low‐quality females (because they are more likely to produce high‐quality offspring).

As predicted by the Trivers‐Willard hypothesis, low‐quality mothers produce relatively more of the sex with lower variance in reproductive success, while high‐quality mothers produce more of the higher variance sex. In this case, the sex ratio of high‐quality mothers favors exclusively males ($s_{2}^{}*\;=\;1$), while the sex ratio of low‐quality mothers is female‐biased ($s_{1}^{}*\;<\;0.5$). When $c_{1}\;=\;c_{2}\;=\;0.5$, *MRVR* = *FRVR* = 1 and equal sex ratios for both $s_{1}^{}*$ and $s_{2}^{}*$ can occur.

#### Female variance in reproductive success

Female variance in reproductive success is affected by the difference between the low‐ and high‐quality female resource investment rates, $R_{1}$ and $R_{2}$ from (37). Again, high‐quality females should have more resources for offspring production ($R_{2}\;>\;R_{1}$).

Figure 8B shows how *MRVR*,* FRVR*, and $s^{}*$ vary with $R_{1}$. We set $R_{2}\;=\;30-R_{1}$, so that the left side of the *x*‐axis corresponds to a high variance in female resource investment ($R_{1}\;=\;0.25,R_{2}\;=\;29.75$), and the right side corresponds to no variance in female resource investment ($R_{1}\;=\;R_{2}\;=\;15$). Thus, *FRVR* is highest on the left and converges to 1 on the right. We assume that high‐ and low‐quality males do not differ ($c_{2}\;=\;c_{1}$), so that *MRVR* = 1 always. In this case, *FRVR* ≥ *MRVR* at all $s^{}*$; that is, females always have the greater variance in reproductive success.

Again, consistent with the Trivers‐Willard effect, high‐quality mothers favor the higher variance sex (females). While high‐quality mothers produce relatively more high variance female offspring ($s_{2}^{}*\;<\;0.5$), low‐quality mothers produce all low variance male offspring ($s_{1}^{}*\;=\;1$). Although low‐quality females are relatively unproductive, all males are equally likely to mate with high‐quality females and produce high‐quality grandchildren. As a result, it appears that low‐quality mothers evolve to maximize their sons.

#### The effect of quality inheritance

Lastly, we consider how $s^{}*$ is affected by the quality inheritance probability $q_{ij}$ in (38). We assume that quality depends only on mothers, which produce offspring of the same quality with a probability $q\;=\;q_{jj}>0.5$. An increase in *q* increases the value of high‐quality mothers, because they are more likely to generate high‐quality offspring. When *q* is high, high‐quality females can become very valuable, leading high‐quality mothers to prefer daughters over sons (Leimar 1996).

We also include both the male advantage and female advantages of high‐quality individuals; that is, high‐quality males are more competitive ($c_{2}\;>\;c_{1}$) * and* high‐quality females are more fertile ($R_{2}\;>\;R_{1}$). Which sex has the larger reproductive variance now varies with *q*.

**Figure 9:**
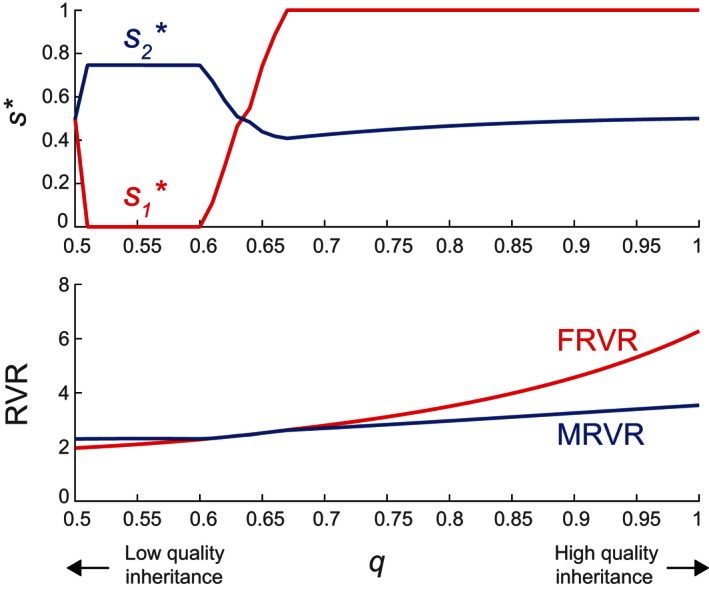
Singular strategies $s^{}*$ ($s_{1}^{}*$ for low‐quality females, $s_{2}^{}*$ for high‐quality females) and reproductive value ratios *MRVR*,* FRVR* as functions of the same quality inheritance probability $q\;=\;q_{jj}$. Both the male advantage ($c_{2}\;>\;c_{1}$) and female advantage ($R_{2}\;>\;R_{1}$) are present, with $c_{1}\;=\;0.1,c_{2}\;=\;0.9$, $R_{1}\;=\;10,R_{2}\;=\;20$. Other model parameters are as given in Table 1.

As shown in Figure 9, at low *q*, the variance in reproductive success of males exceeds that of females (*MRVR* > *FRVR*). High‐quality mothers thus have male‐biased sex ratios; low‐quality mothers, in contrast, produce exclusively daughters. At intermediate *q*, there is an interval where *MRVR* = *FRVR* corresponding to interior SSs of $s^{}*$. At higher *q*, females become the sex with higher variance in reproductive success (*FRVR* > *MRVR*). High‐quality mothers ultimately converge to the equal sex ratio $s_{2}\;=\;0.5$, while low‐quality mothers produce only sons.

These results are consistent with the predictions of the Trivers‐Willard hypothesis, in that high‐quality mothers always produce more of the sex with greater variance in reproductive value than lower quality mothers do. At intermediate values of *q*, however, there is a region where *MRVR* = *FRVR*; this corresponds to interior (Type 1) SSs where both $s_{1}$ and $s_{2}$ are between 0 and 1 (see the subsequent section “Reproductive value ratios and the nature of $s^{}*$”). Because males and females have the same reproductive value ratios at interior SSs, it is not obvious from the Trivers‐Willard hypothesis which sex the high‐quality parents will favor.

#### Reproductive value ratios and the nature of $s^{}*$

In this section, we determine the reproductive value ratios at equilibrium for each type of SS $s^{}*$, and their implications for the Trivers‐Willard hypothesis. Recall that there are five types of $s^{}*$ in this model (an interior point and four boundary equilibria), each of which implies different conditions for the selection gradients (derivatives of invasion fitness) at equilibrium (summarized in Fig. 3). These selection gradients, in turn, depend on the reproductive values at equilibrium (the left eigenvector $v^{\prime }$) through (16). This permits us to invert the calculations presented thus far, which focus on finding $s^{}*$ in various scenarios. We now determine the relationship between male and female reproductive value ratios, given each possible type of $s^{}*$.

In Appendix A, we find the relationship between the male and female reproductive value ratios *MRVR* (44) and *FRVR* (45) at each type of $s^{}*$. These relationships are summarized in Table 3. Because the *RVRs* are measures of the variance in reproductive success for each sex (see the previous section “Variance in reproductive success”), each type of SS therefore also implies a certain relationship between male and female reproductive success. A Type 2 SS, for example, requires that *MRVR* < *FVRV* – that is, the variance in reproductive success of females must exceed that of males.

**Table 3:** Relationships between the male and female reproductive value ratios MRVR and FRVR at each of the five possible singular strategies $s^{}*$ (in Figure 3)

Type of SS	Low‐quality sex ratio ($s_{1}$)	High‐quality sex ratio ($s_{2}$)	Reproductive value ratios	Offspring cost ratios	Examples
1 (interior)	$0\;<\;s_{1}\;<\;1$	$0\;<\;s_{2}\;<\;1$	*MRVR* = *FRVR*	$\frac{C_{m}}{C_{f}}$ = $\frac{v_{m,01}}{v_{f,01}}$ = $\frac{v_{m,02}}{v_{f,02}}$	Figure 9 (mid *q*)
2 (boundary)	$0\;<\;s_{1}\;<\;1$	0 (all females)	*MRVR* < *FRVR*	$\frac{C_{m}}{C_{f}}$ < $\frac{v_{m,01}}{v_{f,01}}$, >$\frac{v_{m,02}}{v_{f,02}}$	Not observed
3 (boundary)	$0\;<\;s_{1}\;<\;1$	1 (all males)	*MRVR* > *FRVR*	$\frac{C_{m}}{C_{f}}$ > $\frac{v_{m,01}}{v_{f,01}}$, <$\frac{v_{m,02}}{v_{f,02}}$	Figure 8A (all $c_{1}$)
4 (boundary)	0 (all females)	$0\;<\;s_{2}\;<\;1$	*MRVR* > *FRVR*	$\frac{C_{m}}{C_{f}}$ > $\frac{v_{m,01}}{v_{f,01}}$, <$\frac{v_{m,02}}{v_{f,02}}$	Figure 9 (low *q*)
5 (boundary)	1 (all males)	$0\;<\;s_{2}\;<\;1$	*MRVR* < *FRVR*	$\frac{C_{m}}{C_{f}}$ < $\frac{v_{m,01}}{v_{f,01}}$, >$\frac{v_{m,02}}{v_{f,02}}$	Figure 8B (all $R_{1}$), Figure 9 (high *q*)

Each of the five types of $s^{}*$ also has a certain biological interpretation (Table 4). At Type 3 and 4 SSs, high‐quality mothers will produce relatively more sons than low‐quality mothers do; at Type 2 and 5 SSs, high‐quality mothers will produce more daughters. This allows us to link variance in reproductive success, as given by *MRVR* and *FRVR*, to the sex favored by high‐quality mothers, as invoked by the Trivers‐Willard hypothesis.

**Table 4:** How the sex preferred by high‐quality mothers corresponds to different sex ratios, types of SS (Figure 3), and reproductive value ratio relationships. The first two cases correspond to the conditions (46) and the second two cases correspond to conditions (47)

High‐quality mothers have more:	Sex ratios	Type of SS	Greater RVR
Sons	$s_{1}\;=\;0\;\;\mathrm{and}\;\;0\;<\;s_{2}\;<\;1$	4	*MRVR*
	$0<s_{1}<1\;\;\mathrm{and}\;\;s_{2}=1$	3	
Daughters	$s_{1}\;=\;1\;\;\mathrm{and}\;\;0\;<\;s_{2}\;<\;1$	5	*FRVR*
	$0\;<\;s_{1}\;<\;1\;\;\mathrm{and}\;\;s_{2}\;=\;0$	2	
Either	$0\;<\;s_{1}\;<\;1\;\;\mathrm{and}\;\;0\;<\;s_{2}\;<\;1$	1	*MRVR*=*FRVR*

As shown in Appendix A, we find that high‐quality mothers consistently favor sons when *MRVR* > *FRVR* (Type 3 or 4 SS) and daughters when *FRVR* > *MRVR* (Type 2 or 5 SS). These results confirm a Trivers‐Willard effect in our model and are similar to those of Leimar's simpler model (1996), which does not consider juvenile or union stages. We also find that when high‐quality mothers produce exclusively one sex (Types 2 and 3 SSs), they always favor the sex with the greater reproductive value ratio. However, when low‐quality mothers produce exclusively one sex (Type 4 and 5 SSs), they always favor the sex with the lower reproductive value ratio.

Our results demonstrate the presence of a “specialization principle” — unless *MRVR* = *FRVR* at equilibrium, one maternal quality will produce all sons or all daughters (i.e., have a Type 2–5 boundary SS). The RVRs are only equal at Type 1 (interior) SSs, where high‐quality mothers may favor either sex. Interior SSs are unique in that they do not experience selective pressure in any direction, because the selection gradients are zero for both sex ratios. This suggests that selective pressure only ceases completely when both male and female reproductive value ratios are equal (*MRVR* = *FRVR*). When *MRVR* = *FRVR*, infinite equilibria also appear in the model of Leimar (1996). Our model does not produce infinite equilibria; the reason for this difference is presently unknown.

Interior $s^{}*$ are also the only type of SS where the ratio of juvenile male to juvenile female reproductive values equals the ratio of the sex‐specific resource costs. Specifically, by (A5):

(48) $$\frac{C_{m}}{C_{f}}=\frac{v_{m,01}}{v_{f,01}}=\frac{v_{m,02}}{v_{f,02}}$$

This result holds true for both low‐quality juveniles ($v_{m,01}/v_{f,01}$) and high‐quality juveniles ($v_{m,02}/v_{f,02}$). A similar result for the SS of a single sex ratio was found in Shyu and Caswell 2016, Case 1). As in the single sex ratio case, this suggests that the sex ratios evolve toward an “equal investment principle,” where the ratio of male to female reproductive values equals to the ratio of the sex‐specific resource costs. If, however, such a point does not exist within the biologically constrained region, $s^{}*$ becomes a boundary SS and equal investment no longer holds (Table 3, “Offspring costs” column).

## Discussion

When a trait like the primary sex ratio varies with an individual's condition, the evolution of that trait may be difficult to anticipate. Because multiple conditions create population structure, and the reproductive advantages of both sexes depends on demographic factors like survival, fecundity, and life span (Leimar 1996; Schwanz et al. 2006), an explicitly demographic model is valuable for understanding facultative sex ratio evolution.

We have developed a new framework for modeling sex ratio evolution that combines three key components: explicit stage structure including multiple sexes and conditions, a nonlinear frequency‐dependent mating process, and evolutionary calculations directly obtained from adaptive dynamics. Each of these three components relaxes one of the limiting assumptions found in the existing sex ratio evolution literature and allows our framework to be adapted to a variety of scenarios. Our models could, for example, be easily extended to include additional population structure in the form of more age classes, physiological conditions, or other kinds of parental differences.

Here, we have presented two specific applications of this framework, each of which includes two maternal conditions with different sex ratios. In these cases, the overall sex ratio strategy **s** is a vector trait with two simultaneously evolving components. Using multidimensional adaptive dynamics, we analyzed both the transient and the long‐term evolution of **s** in cases where individuals differed in age or quality.

In these particular models, **s** displays a wide range of evolutionary behavior. The sex ratio strategy may evolve to an interior SS where both $s_{1}$ and $s_{2}$ are between 0 and 1, or a boundary SS where either $s_{1}$ or $s_{2}$ is 0 or 1 (i.e., mothers of a particular condition produce exclusively one sex). Previous models of facultative sex ratios have similarly found cases where at least one maternal condition only produces offspring of a single sex (e.g., Leimar 1996; Schwanz et al. 2006).

We have also found cases where **s** converges to a line of selectively neutral strategies. This line contains an infinite number of equally viable sex ratio combinations; this may be relevant to why empirical studies have observed so many different, and occasionally contradictory, relationships between sex ratios and maternal conditions (e.g., Jacobsen et al. 1999 in humans, Sheldon and West 2004 in ungulates). Ultimately, our model lends insight into the demographic factors that cause different types of evolutionary singular strategies and, in the case of multiple qualities, their relationships with the reproductive values that underlie the Trivers‐Willard hypothesis.

Although we considered only two conditions at a time (i.e., young and old, high‐ and low‐quality), in reality, individuals will vary across a spectrum of conditions. As alluded to above, our matrix model could be expanded to accommodate more stages for additional conditions, though continuously varying traits and conditions may require an alternative approach. Our model also assumes that mating preferences are proportional to the relative abundances (or weighted abundances) of adult stages, through functions like (10) and (11). Ranking systems where mating preferences depend on the overall composition of the population (e.g. females prefer the largest males currently available) are not explicitly covered by our formulation.

Several other aspects of our model could also be modified to explore different scenarios. We assumed, for example, that the effects of mutations on the sex ratios of younger and older (or low‐ and high‐quality) individuals were uncorrelated. However, a mutation in one gene may affect multiple traits through pleiotropic effects. Antagonistic pleiotropy, whereby selection promotes genes that are beneficial earlier in life, but detrimental later in life, may be an important factor in the development of senescence (Williams 1957). Charnov (1982) hypothesized that this may influence how sex ratios shift with maternal age – that is, factors reducing mortality from early reproduction might increase mortality from later reproduction. While we found changes in age‐specific sex ratios, even without accounting for these kinds of pleiotropic effects, one could explicitly incorporate mutational correlations by modifying the mutational variance matrix **V**(**s**) (20).

Although we have considered only the effects of maternal condition, paternal condition may also influence the primary sex ratio. Paternal attractiveness is of particular interest, in that females mated to attractive males may produce more sons to inherit their father's attractiveness. Resulting sex ratios depend on the nature of the female mating preference and costs and benefits of attractive male traits (Pen and Weissing 2000; Fawcett et al. 2007; West 2009). Paternal age may also affect offspring sex ratios. Several large‐scale studies on human populations, for instance, have found more significant correlations between sex ratios and paternal ages than sex ratios and maternal ages (reviewed in Jacobsen et al. 1999). We previously assumed that any union $u_{ij}$ with a Condition *j* female has sex ratio $s_{j}$; that is, the primary sex ratio depends only on the maternal condition. However, our model could easily be modified to have sex ratios depend on the paternal condition as well.

Lastly, we do not consider any costs or mechanisms for switching between the facultative sex ratios $s_{1}$ and $s_{2}$. Costly sex ratio manipulation, e.g., via selective abortion, may significantly affect sex ratio evolution (Pen and Weissing 2002), and cases where one parental condition uses a very different sex ratio from the other may be less feasible if there are high costs for switching sex ratios. There may also be biological limits to how much the sex ratio can be adjusted. Although actual mechanisms for sex ratio adjustment are still largely unknown, glucose levels *in utero* may be an important factor (Cameron 2004).

## Conflict of Interest

None declared.

## Figures and Tables

**Figure 10:**
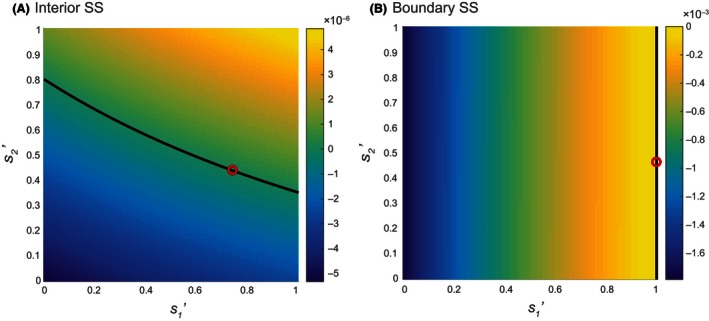
The invasion fitness (15) as a function of the mutant sex ratios $s_{1}^{\prime }$ (low‐quality females) and $s_{2}^{\prime }$ (high‐quality females). The resident population is at its SS sex ratio strategy $s^{}*$. Red dots indicate the location of $s^{}*$, while black lines indicate isoclines of zero invasion fitness (where the mutant and resident are equally fit). Plots are for the Case 2 (maternal quality) model with different values of *q* (same as Figure 9). $C_{m}\;=\;C_{f}\;=\;0.5,c_{1}\;=\;0.1,c_{2}\;=\;0.9,R_{1}\;=\;10,R_{2}\;=\;20$; other parameters are as given in Table 1. (A) Example of an interior SS (Type 1) for *q* = 0.65. The eigenvalues of **H** are $-1.6\;\times \;10^{-6}$ and $2.9\;\times \;10^{-7}$. (B) Example of a boundary SS (Type 5) for *q* = 0.8. The eigenvalues of **H** are $-8.5\;\times \;10^{-4}$ and $2\;\times \;10^{-7}$.

**Figure 11:**
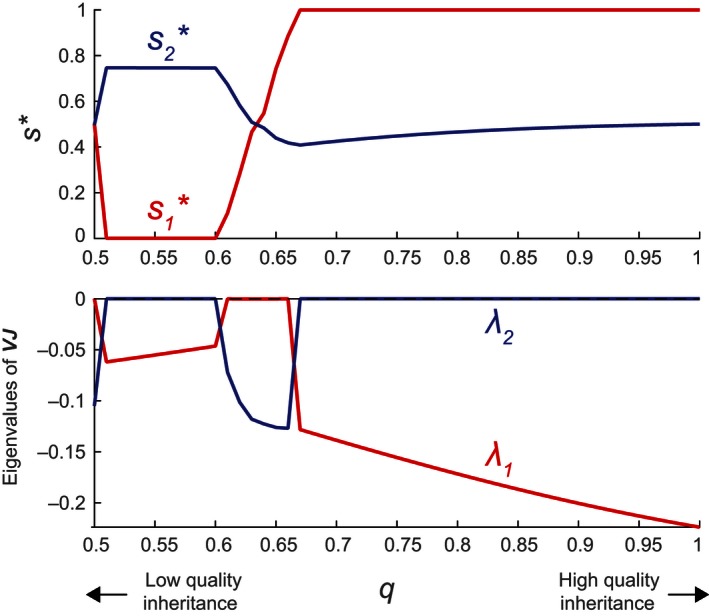
Eigenvalues $\lambda_{1},\lambda_{2}$ of the matrix **VJ**, which is used to characterize convergence stability (Table 5). Model is the same as in Figure 11.

## Appendix A Reproductive Value Ratios at Singular Strategies

In this section, we calculate the relation between *MRVR* and *FRVR* at each of the five types of singular strategies $s^{}*$ in Figure 3. All SSs occur when one or both components of the selection gradient (17) is 0. As shown in (16), the selection gradient depends on the mutant reproductive value vector $v^{\prime }$.

Given a *s*×1 population vector, the first term in (16) is the $1\times s^{2}$ vector:

(A1) $$\begin{array}{c} {w^{\prime }}^{⊺}⊗{v^{\prime }}^{⊺}=\left(\begin{array}{ccccccccc} w_{1}v_{1} & w_{1}v_{2} & \cdots & w_{1}v_{s} & \left|\cdots \right| & w_{s}v_{1} & w_{s}v_{2} & \cdots & w_{s}v_{s} \end{array}\right) \end{array}$$

where $w_{i}$ is the i${}^{\mathrm{th}}$ entry of $w^{\prime }$ (stable stage frequency of stage *i*), and $v_{i}$ is the i${}^{\mathrm{th}}$ entry of $v^{\prime }$ (reproductive value of stage *i*).

The second term in (16) is the $s^{2}\times 2$ vector:

(A2) $$\frac{d\text{vec}A^{\prime }}{ds^{\prime }}=\frac{1}{3}\left(\frac{d\text{vec}T^{\prime }}{ds^{\prime }}+\frac{d\text{vec}B^{\prime }}{ds^{\prime }}+\frac{d\text{vec}U^{\prime }}{ds^{\prime }}\right)$$

We will use (42), (39), and (43) for the rate matrices **U**,** B**, and **T**, respectively.

After substituting (A1) and (A2) into (16) and simplifying the results, we obtain the following expressions for the components of the selection gradient:

(A3) $$\begin{array}{cc} {\frac{d\lambda^{\prime }}{{ds}^{\prime }}}^{⊺} & =\left(\begin{array}{c} \frac{\partial \lambda^{\prime }}{\partial s_{1}^{\prime }}{|}_{s^{\prime }=s}\\ \frac{\partial \lambda^{\prime }}{\partial s_{2}^{\prime }}{|}_{s^{\prime }=s} \end{array}\right)\\ & =\left(\begin{array}{cc} C_{1} & 0\\ 0 & C_{2} \end{array}\right)\left(\begin{array}{c} -C_{m}\left[qv_{f,01}+\left(1-q\right)v_{f,02}\right]+C_{f}\left[qv_{m,01}+\left(1-q\right)v_{m,02}\right]\\ -C_{m}\left[\left(1-q\right)v_{f,01}+qv_{f,02}\right]+C_{f}\left[\left(1-q\right)v_{m,01}+qv_{m,02}\right] \end{array}\right) \end{array}$$

where $C_{1}$ and $C_{2}$ are positive quantities that do not affect the signs or zeroes of the selection gradients.

At each type of SS, one or both components of the selection gradient (A3) will be 0. We will examine each of the five type of SS from Figure 3 to determine their corresponding reproductive value ratios.

### Interior SS (Type 1)

For an interior SS, both components of the selection gradient (A3) are simultaneously 0. Thus, when evaluated at the SS:

(A4) $$\begin{array}{cc} 0 & =-C_{m}\left[qv_{f,01}+\left(1-q\right)v_{f,02}\right]+C_{f}\left[qv_{m,01}+\left(1-q\right)v_{m,02}\right]\\ 0 & =-C_{m}\left[\left(1-q\right)v_{f,01}+qv_{f,02}\right]+C_{f}\left[\left(1-q\right)v_{m,01}+qv_{m,02}\right] \end{array}$$

Solving for the reproductive values in (A4), we obtain the following equalities:

(A5) $$\frac{C_{m}}{C_{f}}=\frac{v_{m,01}}{v_{f,01}}=\frac{v_{m,02}}{v_{f,02}}$$

From (A5), we also see that:

(A6) $$\begin{array}{cc} \frac{v_{m,02}}{v_{m,01}} & =\frac{v_{f,02}}{v_{f,01}}\\ MRVR & =FRVR \end{array}$$

That is, the male and female reproductive value ratios are equal at any interior SS.

#### Boundary SS (Type 2–5)

For a boundary SS, only one component of the selection gradient (A3) is 0. The other component is either positive or negative depending on the specific type of boundary SS (Fig. 3).

- For a Type 2 SS, $\frac{d\lambda^{\prime }}{ds_{1}^{\prime }}=0$. From (A3), we see that:
  (A7) $$C_{m}\left[qv_{f,01}+\left(1-q\right)v_{f,02}\right]=C_{f}\left[qv_{m,01}+\left(1-q\right)v_{m,02}\right]$$
  Also for a Type 2 SS, $\frac{d\lambda^{\prime }}{ds_{2}^{\prime }}\;<\;0$. Solving (A7) for $v_{m,01}$ or $v_{m,02}$ and substituting the result into the expression for $\frac{d\lambda^{\prime }}{ds_{2}^{\prime }}$ in (A3), we obtain:
  (A8) $$\begin{array}{cc} \frac{d\lambda^{\prime }}{ds_{2}^{\prime }} & =\frac{2q-1}{q}\left(C_{f}v_{m,02}-C_{m}v_{f,02}\right)\\ & =\frac{2q-1}{q-1}\left(C_{f}v_{m,01}-C_{m}v_{f,01}\right)\\ & <0 \end{array}$$
  Because 0.5 ≤ *q* ≤ 1, the conditions in (A8) become:
  (A9) $$\begin{array}{cc} \frac{v_{m,02}}{v_{f,02}} & <\frac{C_{m}}{C_{f}}\\ \frac{v_{m,01}}{v_{f,01}} & >\frac{C_{m}}{C_{f}} \end{array}$$
  Combining the two inequalities in (A9) and noting that all the quantities are positive, we obtain:
  (A10) $$\begin{array}{cc} \frac{v_{m,02}}{v_{m,01}} & <\frac{v_{f,02}}{v_{f,01}}\\ MRVR & <FRVR \end{array}$$
- For a Type 3 SS, $\frac{d\lambda^{\prime }}{ds_{1}^{\prime }}=0$ as well, but $\frac{d\lambda^{\prime }}{ds_{2}^{\prime }}>0$. We accordingly apply (A9) with the inequalities flipped to obtain:
  (A11) $$\begin{array}{cc} \frac{v_{m,02}}{v_{m,01}} & >\frac{v_{f,02}}{v_{f,01}}\\ MRVR & >FRVR \end{array}$$
- For a Type 4 SS, $\frac{d\lambda^{\prime }}{ds_{2}^{\prime }}=0$. From (A3), we see that:
  (A12) $$C_{m}\left[\left(1-q\right)v_{f,01}+qv_{f,02}\right]=C_{f}\left[\left(1-q\right)v_{m,01}+qv_{m,02}\right]$$
  Also for a Type 4 SS, $\frac{d\lambda^{\prime }}{ds_{1}^{\prime }}<0$. Using methods analogous to those above, it can be shown that:
  (A13) $$\begin{array}{cc} \frac{v_{m,02}}{v_{m,01}} & >\frac{v_{f,02}}{v_{f,01}}\\ MRVR & >FRVR \end{array}$$
- For a Type 5 SS, $\frac{d\lambda^{\prime }}{ds_{2}^{\prime }}\;=\;0$ and $\frac{d\lambda^{\prime }}{ds_{1}^{\prime }}\;>\;0$, which yields:
  (A14) $$\begin{array}{cc} \frac{v_{m,02}}{v_{m,01}} & <\frac{v_{f,02}}{v_{f,01}}\\ MRVR & <FRVR \end{array}$$

All these results are summarized in Table 3.

## Appendix B Stability of 2D Singular Strategies

The evolutionary and convergence stability of a singular strategy is characterized using the local second derivatives of the invasion fitness (15). We have previously showed second derivatives calculations for a single evolving sex ratio in Shyu and Caswell (2016). Although analogous calculations can be performed for vector‐valued traits, the SS stability conditions are more stringent (Table 5).

**Table 5:** Conditions for the evolutionary and convergence stability of a vector‐valued singular strategy. These conditions depend on the Hessian **H** of the invasion fitness (B1), the Jacobian **J** of the selection gradient (B2), and the mutational variance–covariance matrix **V** in the canonical equation (18) (Apaloo and Butler 2009; Leimar 2009)

Type of stability	Sufficient condition for stability	Sufficient condition for instability
Evolutionary stability	**H** is negative definite	**H** is positive definite
Convergence stability	**VJ** has only eigenvalues with negative real parts	**VJ** has at least one eigenvalue with a positive real part
Strong convergence stability	**J** is negative definite	**J** is positive definite

### Evolutionary stability

Evolutionary stability indicates that the SS cannot be invaded by any nearby mutants. It depends on **H**, the Hessian matrix of the invasion fitness:

(B1) $$H=\frac{\partial^{2}\lambda^{\prime }}{{\partial s}^{\prime }\partial {s^{\prime }}^{⊺}}$$

This expression can be calculated using the matrix calculus methods detailed in Shyu and Caswell 2016, section 3.3.1).

A SS $s^{}*$ is evolutionarily stable if **H** is negative definite at $s^{}*$ (Apaloo and Butler 2009). Many of the **H** matrices in our model are negative semidefinite or indefinite because of zero eigenvalues, leading to inconclusive stability results. Figure 10 shows several examples of the corresponding invasion fitness landscapes. In these cases, and many others in our model, $s^{}*$ lies on a line of points with zero invasion fitness. This means that there are an infinite number of mutant sex ratio combinations that have the same fitness as $s^{}*$.

This is similar to our results for a single evolving sex ratio (Shyu and Caswell 2016), where $s^{}*$ lies on a zero isocline for which any mutant sex ratios have equal fitness. This type of SS is known as a selectively neutral or weak form ESS (Uyenoyama and Bengtsson 1979; Bull and Charnov 1988). Once the population reaches a weak form ESS, there is no selective pressure to evolve further, because no mutants have positive invasion fitness. However, since all mutants have the same fitness as the resident, they may potentially coexist at low levels.

#### Convergence stability

Convergence stability indicates that the SS is an evolutionary attractor that the trait will converge to through small mutations. It depends on **J**, the Jacobian matrix of the selection gradient (Leimar 2009):

(B2) $$J=H+\frac{\partial^{2}\left(\lambda^{\prime }-\lambda \right)}{{\partial s}^{\prime }\partial s^{⊺}}$$

Again, this expression can be calculated using the matrix calculus methods detailed in Shyu and Caswell 2016, section 3.3.2)

A SS $s^{}*$ is convergence stable if **VJ**, the product of the mutational matrix from (18) and the Jacobian (B2), has eigenvalues with negative real parts at $s^{}*$. The SS is strongly convergence stable (stable for any smooth, symmetric, positive definite **V**) if **J** is negative definite at $s^{}*$ (Leimar 2009). Again, many of the **VJ** and **J** matrices in our model are negative semidefinite or indefinite because of zero eigenvalues, leading to inconclusive stability results. Stability can be especially difficult to characterize for boundary SSs, which have limited directions for evolution.

Example eigenvalues of **VJ** are shown in Figure 11. Note that for boundary SSs, at least one diagonal entry of **V**, as given by (20), will be 0, resulting in zero eigenvalues of **VJ**. Zero eigenvalues appear to correspond to eigenvectors pointing toward the boundaries, while negative eigenvalues correspond to eigenvectors pointing along the boundaries.
